# Fast Proxy Centers for the Jeffreys Centroid: The Jeffreys–Fisher–Rao Center and the Gauss–Bregman Inductive Center

**DOI:** 10.3390/e26121008

**Published:** 2024-11-22

**Authors:** Frank Nielsen

**Affiliations:** Sony Computer Science Laboratories, Tokyo 141-0022, Japan; frank.nielsen.x@gmail.com

**Keywords:** Kullback–Leibler divergence, exponential family, Bregman divergence, quasi-arithmetic mean, Fisher–Rao geodesic, information geometry, Lambert *W* function, geometric optimization

## Abstract

The symmetric Kullback–Leibler centroid, also called the Jeffreys centroid, of a set of mutually absolutely continuous probability distributions on a measure space provides a notion of centrality which has proven useful in many tasks, including information retrieval, information fusion, and clustering. However, the Jeffreys centroid is not available in closed form for sets of categorical or multivariate normal distributions, two widely used statistical models, and thus needs to be approximated numerically in practice. In this paper, we first propose the new Jeffreys–Fisher–Rao center defined as the Fisher–Rao midpoint of the sided Kullback–Leibler centroids as a plug-in replacement of the Jeffreys centroid. This Jeffreys–Fisher–Rao center admits a generic formula for uni-parameter exponential family distributions and a closed-form formula for categorical and multivariate normal distributions; it matches exactly the Jeffreys centroid for same-mean normal distributions and is experimentally observed in practice to be close to the Jeffreys centroid. Second, we define a new type of inductive center generalizing the principle of the Gauss arithmetic–geometric double sequence mean for pairs of densities of any given exponential family. This new Gauss–Bregman center is shown experimentally to approximate very well the Jeffreys centroid and is suggested to be used as a replacement for the Jeffreys centroid when the Jeffreys–Fisher–Rao center is not available in closed form. Furthermore, this inductive center always converges and matches the Jeffreys centroid for sets of same-mean normal distributions. We report on our experiments, which first demonstrate how well the closed-form formula of the Jeffreys–Fisher–Rao center for categorical distributions approximates the costly numerical Jeffreys centroid, which relies on the Lambert *W* function, and second show the fast convergence of the Gauss–Bregman double sequences, which can approximate closely the Jeffreys centroid when truncated to a first few iterations. Finally, we conclude this work by reinterpreting these fast proxy Jeffreys–Fisher–Rao and Gauss–Bregman centers of Jeffreys centroids under the lens of dually flat spaces in information geometry.

## 1. Introduction

Let (X,F) be a measurable space with sample space X and σ-algebra of events F, and μ a positive measure. We consider a finite set $\left\{P_{1},\ldots ,P_{n}\right\}$ of *n* probability distributions all dominated by μ and weighted by a vector *w* belonging to the open standard simplex $\Delta_{n}=\left\{x=\left(x_{1},\ldots ,x_{n}\right)\;:\;x_{1}>0,\ldots ,x_{n}>0,\sum_{i=1}^{n}x_{i}=1\right\}\subset R^{n}$. Let $P=\{p_{1},\ldots ,p_{n}\}$ be the Radon–Nikodym densities of $P_{1},\ldots ,P_{n}$ with respect to μ, i.e., $p_{i}=\frac{dP_{i}}{d\mu }$.

The Kullback–Leibler divergence (KLD) between two densities p(x) and q(x) is defined by $D_{\mathrm{KL}}\left(p:q\right)=\int p\left(x\right)\log\frac{p(x)}{q(x)}\;d\mu \left(x\right)$. The KLD is asymmetric: $D_{\mathrm{KL}}\left(p:q\right)\neq D_{\mathrm{KL}}\left(q:p\right)$. We use the argument delimiter ‘:’ as a notation to indicate this asymmetry. The Jeffreys divergence [1] symmetrizes the KLD as follows:

$$\begin{array}{ccc} D_{J}\left(p,q\right) & = & D_{\mathrm{KL}}\left(p:q\right)+D_{\mathrm{KL}}\left(q:p\right),\\ & = & \int_{X}\left(p\left(x\right)-q\left(x\right)\right)\log\frac{p(x)}{q(x)}d\mu \left(x\right). \end{array}$$

In general, the *D*-barycenter $C_{D}$ of P with respect to a statistical dissimilarity measure D(·:·) yields a notion of centrality $C_{R}$ defined by the following optimization problem:

(1) $$c_{R}=\arg\min_{p}\sum_{i=1}^{n}w_{i}\;D\left(p_{i}:p\right).$$

Here, the upper case letter ‘R’ indicates that the optimization defining the *D*-barycenter is carried on the right argument. When $w=(\frac{1}{n},\ldots ,\frac{1}{n})$ is the uniform weight vector, the *D*-barycenter is called the *D*-centroid. We shall loosely call centroids barycenters in the remainder even when the weight vector is not uniform. Centroids with respect to information-theoretic measures have been studied in the literature.

Let us mention some examples of centroids: The entropic centroids [2] (i.e., Bregman centroids and *f*-divergences centroids), the Burbea–Rao and Bhattacharyya centroids [3], the α-centroids with respect to α-divergences [4], the Jensen–Shannon centroids [5], etc.

The $D_{J}$-centroid is also called the symmetric Kullback–Leibler (SKL) divergence centroid [6] in the literature. However, since there are many possible symmetrizations of the KLD [7] like the Jensen–Shannon divergence [8] or the resistor KLD [9], we prefer to use the term Jeffreys centroid instead of SKL centroid to avoid any possible ambiguity on the underlying divergence. Notice that the square root of the Jensen–Shannon divergence is a metric distance [10,11] but all powers $D_{J}^{\alpha }$ of Jeffreys divergence $D_{J}$ for α>0 do not yield metric distances [12].

This paper considers the Jeffreys centroids of a finite weighted set of densities $P=\{p_{\theta_{1}},\ldots ,p_{\theta_{n}}\}$ belonging to some prescribed exponential family [13] E:

(2) $$c=\arg\min_{p}\sum_{i=1}^{n}w_{i}\;D_{J}\left(p_{\theta_{i}},p\right).$$

In particular, we are interested in computing the Jeffreys centroids for sets of categorical distributions or sets of multivariate normal distributions [14].

**Figure 1:**
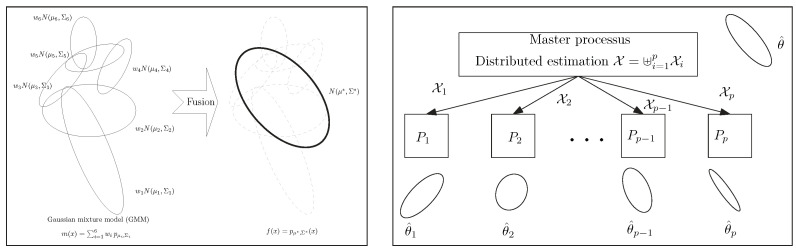
Application of centroids and centers in signal processing. (**Left**): information fusion and mixture model simplification, a 2D Gaussian mixture model (GMM) is simplified to a single bivariate normal distribution. (**Right**): distributed estimation, a data set is split among *p* processes $P_{i}$s, which first estimate the statistical model parameters ${\hat{\theta }}_{i}$s. Then, the processus models are aggregated to yield a single consolidated model $\hat{\theta }$.

In general, centroids are used in *k* means [15,16]-type clustering or hierarchical clustering (e.g., Ward criterion [17]) and information fusion tasks [18] (related to distributed model estimation [19]) among others. See Figure 1. The choice of the dissimilarity measure depends on the application at hand [20]. Clustering with respect to Jeffreys divergence/-Jeffreys centroid has proven useful in many scenarios: for example, it was shown to perform experimentally better than Euclidean or square Euclidean distances for compressed histograms of gradient descriptors [21] or in fuzzy clustering [22]. Jeffreys divergence has also been used for image processing [23], including image segmentation [24], speech processing [25], and computer vision [26], just to name a few. In particular, finding weighted means of centered 3D normal distributions plays an important role in diffusion tensor imaging (DTI) for smoothing and filtering DT images [27] which consist of sets of normal distributions centered at 3D grid locations.

In general, the Jeffreys centroid is not known in closed form for exponential families [28] like the family of categorical distributions or the family of normal distributions often met in applications and thus needs to be numerically approximated in practice. The main contribution of this paper is to present and study two proxy centers as drop-in replacements of the Jeffreys centroids in applications and report the generic structural formula for generic exponential families with an explicit closed-form formula for the families of categorical and multivariate normal distributions. Namely, we define the Jeffreys–Fisher–Rao (JFR) center (Definition 2) and the Gauss–Bregman (GB) inductive center (Definition 3) in Section 2.

This paper is organized as follows: By interpreting in two different ways the closed-form formula of the Jeffreys centroids for the particular case of sets of centered multivariate normal distributions [29] (proof reported in Appendix B), we define the Gauss–Bregman (GB) centers and the Jeffreys–Fisher–Rao (JFR) centers for sets of densities belonging to an exponential family in Section 2. The Jeffreys centroid coincides with both the Gauss–Bregman inductive center and the Jeffreys–Fisher–Rao center for centered multivariate normal distributions but differ from each other in general. In Section 2.4, we study the Gauss–Bregman inductive center [30] induced by the cumulant function of an exponential family and prove the convergence under the separability condition of the generalized Gauss double sequences in the limit (Theorem 3). This Gauss–Bregman center can be easily approximated by limiting the number of iterations of a double sequence inducing it. In Section 4, we report the generic formula for Jeffreys–Fisher–Rao centers for sets of uni-order exponential families [13] and explicitly give the closed-form formula for the categorical family and the multivariate normal family. A comparison of those proxy centers with the numerical Jeffreys centroids is experimentally studied and visually illustrated with some examples. Thus, we propose to use in applications (e.g., clustering) either the fast Jeffreys–Fisher–Rao center when a closed-form formula is available for the family of distributions at hand or the Gauss–Bregman center approximation with a prescribed number of iterations as a drop-in replacement of the numerical Jeffreys centroids while keeping the Jeffreys divergence. Some experiments of the JFR and GB centers are reported for the Jeffreys centroid of categorical distributions in Section 5. Finally, we conclude this paper in Section 6 with a discussion and a generalization of our results to the more general setting of dually flat spaces of information geometry [14].

The core of this paper is followed by an Appendix section as follows: In Appendix A, we explicitly give the algorithm outlined in [31] for numerically computing the Jeffreys centroid of sets of categorical distributions. In Appendix B, we report a proof on the closed-form formula of the Jeffreys centroid for centered normal distributions [29] that motivated this paper. In Appendix C, we explain how to calculate in practice the elaborated closed-form formula for the Fisher–Rao geodesic midpoint between two multivariate normal distributions [32].

## 2. Proxy Centers for Jeffreys Centroids

### 2.1. Background on Jeffreys Centroids

A density $p_{\theta }$ belonging to an exponential family [13] E can be expressed canonically as $p_{\theta }\left(x\right)=\exp\left(\langle \theta ,t(x)\rangle -F\left(\theta \right)\right)\;d\mu \left(x\right)$, where t(x) is a sufficient statistic vector, F(θ)=log∫exp(〈θ,t(x)〉)dμ(x) is the log-normalizer, and θ is the natural parameter belonging to the natural parameter space Θ. We consider minimal regular exponential families [13] like the discrete family of categorical distributions (i.e., μ is the counting measure) or the continuous family of multivariate normal distributions (i.e., μ is the Lebesgue measure).

The Jeffreys centroid of categorical distributions was first studied by Veldhuis [6], who designed a numerical two-nested loops Newton-like algorithm [6]. A random variable *X* following a categorical distribution Cat(p) for a parameter $p\in \Delta_{d}$ in sample space $X=\{\omega_{1},\ldots ,\omega_{d}\}$ is such that $\mathrm{Pr}\left(X=\omega_{i}\right)=p_{i}$. Categorical distributions are often used in image processing to statistically model normalized histograms with non-empty bins. The exact characterization of the Jeffreys centroid was given in [31].

We summarize the results regarding the categorical Jeffreys centroid [31] in the following theorem:

**Theorem** **1** (Categorical Jeffreys centroid [31])**.** *The Jeffreys centroid of a set of n categorical distributions parameterized by $P=\left\{p_{1},\ldots ,p_{n}\right\}\in \Delta_{d}$ arranged in a matrix $P=\left[p_{i,j}\right]\in R^{n\times d}$ and weighted by a vector $w=\left(w_{1},\ldots ,w_{n}\right)\in \Delta^{n}$ is $c\left(\lambda \right)=(c_{1}\left(\lambda \right),\ldots ,c_{d}\left(\lambda \right))$ with*

$$c_{j}\left(\lambda \right)=\frac{a_{j}}{W_{0}\left(\frac{a_{j}}{g_{j}}\;e^{1+\lambda }\right)},\;\forall j\in \left\{1,\ldots ,d\right\},$$

*where $a_{j}=\sum_{i=1}^{n}w_{i}p_{i,j}$ and $g_{j}=\frac{\prod_{i=1}^{n}p_{i,j}^{w_{i}}}{\sum_{j=1}^{d}\prod_{i=1}^{n}p_{i,j}^{w_{i}}}$ are the j-th components of the weighted arithmetic and normalized geometric means, respectively; $W_{0}$ is the principal branch of the Lambert W function [33]; and λ≤0 is the unique real value such that $\lambda =-D_{\mathrm{KL}}\left(c\left(\lambda \right):g\right)$.*

Furthermore, a simple bisection search is reported in [31] §III.B that we convert into Algorithm A1 in Appendix A, which allows one to numerically approximate the Jeffreys centroid to arbitrary fine precision.

### 2.2. Jeffreys Centroids on Exponential Family Densities: Symmetrized Bregman Centroids

The Jeffreys divergence between two densities of an exponential family $E=\{p_{\theta }\left(x\right)=\exp\left(\langle t(x),\theta \rangle -F\left(\theta \right)\right)\;:\;\theta \in \Theta \}$ with cumulant function F(θ) amounts to a symmetrized Bregman divergence [28] (SBD):

$$D_{J}\left(p_{\theta },p_{\theta^{\prime }}\right)=S_{F}\left(\theta ,\theta^{\prime }\right):=\langle \theta_{1}-\theta_{2},\nabla F\left(\theta_{1}\right)-\nabla F\left(\theta_{2}\right)\rangle .$$

Using convex duality, we have $S_{F}\left(\theta ,\theta^{\prime }\right)=S_{F^{*}}\left(\eta ,\eta^{\prime }\right)$, where η=∇F(θ) and $F^{*}\left(\eta \right)=\langle \eta ,{\left(\nabla F\right)}^{-1}\left(\eta \right)\rangle -F\left({\left(\nabla F\right)}^{-1}\left(\eta \right)\right)$ is the Legendre–Fenchel convex conjugate. Thus, the Jeffreys barycenter of $P=\{p_{\theta_{1}},\ldots ,p_{\theta_{n}}\}$ amounts to either a symmetrized Bregman barycenter on the natural parameters $P_{\theta }=\left\{\theta_{1},\ldots ,\theta_{n}\right\}$ with respect to $S_{F}$ or a symmetrized Bregman barycenter on the dual moment parameters $P_{\eta }=\left\{\eta_{1},\ldots ,\eta_{n}\right\}$ with respect to $S_{F^{*}}$.

It was shown in [28] that the symmetrized Bregman barycenter $\theta_{S}$ of *n* weighted points amounts to the following minimization problem involving only the sided Bregman centroids:

(3) $$\begin{array}{ccc} \theta_{S} & := & \arg\min_{\theta \in \Theta }\sum_{i}w_{i}S_{F}\left(\theta ,\theta_{i}\right),\\ \; & \equiv & \arg\min_{\theta \in \Theta }B_{F}\left(\bar{\theta }:\theta \right)+B_{F}\left(\theta :\underline{\theta }\right), \end{array}$$

where $\bar{\theta }=\sum_{i}w_{i}\theta_{i}$ (right Bregman centroid) and $\underline{\theta }={\left(\nabla F\right)}^{-1}\left(\sum_{i}w_{i}\nabla F\left(\theta_{i}\right)\right)$ (left Bregman centroid). Those $\bar{\theta }$ and $\underline{\theta }$ centers are centroids [28] with respect to the Bregman divergence $B_{F}\left(\theta_{1}:\theta_{2}\right)=F\left(\theta_{1}\right)-F\left(\theta_{2}\right)-\langle \theta_{1}-\theta_{2},\nabla F\left(\theta_{2}\right)\rangle$ and reverse Bregman divergence: ${B_{F}}^{*}\left(\theta_{1}:\theta_{2}\right):=B_{F}\left(\theta_{2}:\theta_{1}\right)$:

$$\begin{array}{ccc} \bar{\theta } & = & \arg\min_{\theta }\sum_{i}w_{i}B_{F}\left(\theta_{i}:\theta \right),\\ \underline{\theta } & = & \arg\min_{\theta }\sum_{i}w_{i}B_{F}\left(\theta :\theta_{i}\right)=\arg\min_{\theta }\sum_{i}w_{i}{B_{F}}^{*}\left(\theta_{i}:\theta \right). \end{array}$$

In general, when $H:R^{m}\to R$ is a strictly convex differentiable real-valued function of Legendre type [34], the gradient ∇H is globally invertible (in general, the implicit inverse function theorem only locally guarantees the inverse function) and we can define a quasi-arithmetic center of a point set $P=\{\theta_{1},\ldots ,\theta_{n}\}$ weighted by *w* as follows:

**Definition** **1** (Quasi-arithmetic center)**.** *Let H=∇F be the gradient of a strictly convex or concave differentiable real-valued function F of Legendre type. The quasi-arithmetic center $c_{H}\left(\theta_{1},\ldots ,\theta_{n};w\right)$ is defined by*

$$c_{H}\left(\theta_{1},\ldots ,\theta_{n};w\right)=H^{-1}\left(\sum_{i=1}^{n}w_{i}H\left(\theta_{i}\right)\right).$$

This definition generalizes the scalar quasi-arithmetic means [35] for univariate functions *h*, which are continuous and strictly monotone. Quasi-arithmetic means (QAMs) are also called *f* means or Kolmogorov–Nagumo means. Let $m_{\nabla F}\left(\theta_{1},\theta_{2}\right)=c_{\nabla F}\left(\theta_{1},\theta_{2};\frac{1}{2},\frac{1}{2}\right)$. Notice that $A\left(\theta_{1},\theta_{2}\right)=\nabla F^{*}\left(m_{\nabla F^{*}}\left(\eta_{1},\eta_{2}\right)\right)$ and $A\left(\eta_{1},\eta_{2}\right)=\nabla F(m_{\nabla F}\left(\theta_{1},\theta_{2}\right))$. That is, the arithmetic mean in a primal representation amounts to a QAM in the dual representation.

Thus, we can solve for $\theta_{S}$ by setting the gradient of $L\left(\theta \right)=B_{F}\left(\bar{\theta }:\theta \right)+B_{F}\left(\theta :\underline{\theta }\right)$ to zero. In general, no closed-form formula is known for the symmetrized Bregman centroids, and a numerical approximation method was reported in [28]. To circumvent the lack of a closed-form formula of symmetrized Bregman centroids for clustering, Nock et al. [36] proposed a mixed Bregman clustering where each cluster has two representative dual Bregman centroids $\bar{\theta }=\sum_{i}w_{i}\theta_{i}$ (right Bregman centroid) and $\underline{\theta }={\left(\nabla F\right)}^{-1}\left(\sum_{i}w_{i}\nabla F\left(\theta_{i}\right)\right)$ (left Bregman centroid), and the dissimilarity measure is a mixed Bregman divergence defined by

$$\Delta_{F}\left(\theta_{1}:\theta :\theta_{2}\right):=\frac{1}{2}B_{F}\left(\theta_{1}:\theta \right)+\frac{1}{2}B_{F}\left(\theta :\theta_{2}\right).$$

Notice that minimizing Equation (3) amounts to minimizing the mixed Bregman divergence:

$$\min_{\theta }\Delta_{F}\left(\bar{\theta }:\theta :\underline{\theta }\right).$$

By using the dual parameterization η=∇F(θ) (with dual domain H={∇F(θ):θ∈Θ}) and the dual Bregman divergence $B_{F^{*}}\left(\eta_{1}:\eta_{2}\right)=F^{*}\left(\eta_{1}\right)-F^{*}\left(\eta_{2}\right)-{\langle \eta_{1}-\eta_{2},\nabla F\rangle }^{*}\left(\eta_{1}\right)=B_{F}\left(\theta_{2}:\eta_{1}\right)$, we have

(4) $$\begin{array}{ccc} \theta_{S} & := & \arg\min_{\theta \in \Theta }\sum_{i}w_{i}S_{F}\left(\theta ,\theta_{i}\right),\\ \eta_{S} & = & \arg\min_{\eta \in H}\sum_{i}w_{i}S_{F^{*}}\left(\eta ,\eta_{i}\right),\\ \; & \equiv & \arg\min_{\eta \in H}B_{F^{*}}\left(\eta :\nabla F\left(\bar{\theta }\right)\right)+B_{F^{*}}\left(\nabla F\left(\underline{\theta }\right):\eta \right). \end{array}$$

Since $\nabla F\left(\bar{\theta }\right)={\left(\nabla F^{*}\right)}^{-1}\left(\sum_{i}w_{i}\nabla F^{*}\left(\eta_{i}\right)\right)=\underline{\eta }$ and $\nabla F\left(\underline{\theta }\right)=\nabla F\left({\left(\nabla F\right)}^{-1}\sum_{i}w_{i}\eta_{i}\right)=\bar{\eta }$, we obtain the dual equivalent optimization problem:

$$\theta_{S}=\nabla F^{*}\left(\eta_{S}\right)=\arg\min_{\theta \in \Theta }B_{F}\left(\bar{\theta }:\theta \right)+B_{F}\left(\theta :\underline{\theta }\right),$$

or

$$\eta_{S}=\nabla F\left(\theta_{S}\right)=\arg\min_{\eta \in H}B_{F^{*}}\left(\bar{\eta }:\theta \right)+B_{F^{*}}\left(\eta :\underline{\eta }\right).$$

However, a remarkable special case is the family of multivariate normal distributions centered at the origin for which the Jeffreys centroid was reported in closed form in [29]. Let $N_{0}=\left\{p_{\sum }\;:\;\sum \in {\mathrm{Sym}}^{++}\left(R,d\right)\right\}$ be the exponential family with sufficient statistics $t\left(x\right)=-\frac{1}{2}\left(x,xx^{⊤}\right)$, natural parameter $\theta =\sum^{-1}$ (the precision matrix) where the covariance matrix belongs to the cone ${\mathrm{Sym}}^{++}\left(R,d\right)$ of symmetric positive-definite matrices, inner product 〈X,Y〉=tr(XY), and $F\left(\theta \right)=-\frac{1}{2}\log\det\left(\theta \right)$. In that case, the Jeffreys divergence amounts to a symmetrized Bregman log-det (ld) divergence between the corresponding natural parameters:

$$D_{J}\left(p_{\sum },p_{\sum^{\prime }}\right)=\frac{1}{2}\;\mathrm{tr}(({\sum^{\prime }}^{-1}-\sum^{-1})(\sum -\sum^{\prime }))=:\frac{1}{2}S_{\mathrm{ld}}\left(\sum^{-1},{\sum^{\prime }}^{-1}\right).$$

Using the standard covariance matrix parameterization ∑, we can further express the Jeffreys divergence between two multivariate normal distributions $p_{\sum }$ and $p_{\sum^{\prime }}$ as

$$D_{J}\left(p_{\sum },p_{\sum^{\prime }}\right)=\sum_{i=1}^{d}{\left(\sqrt{\lambda_{i}}-\frac{1}{\sqrt{\lambda_{i}}}\right)}^{2},$$

where $\lambda_{i}$s are the eigenvalues of $\sum^{-1}\sum^{\prime }$. The symmetrized log-det divergence $S_{\mathrm{ld}}$ is also called the symmetrized Stein loss [37,38]. When d=1, this divergence is the symmetrized Itakura–Saito divergence also called the COSH distance [28]**.** The Jeffreys centroid can be characterized using the Fisher–Rao geometry [39] of $N_{0}$ as the Fisher–Rao geodesic midpoint of the sided Kullback–Leibler centroids as follows:

**Theorem** **2** ([29])**.** *The Jeffreys centroid C of a set of n centered multivariate normal distributions $P=\{p_{\sum_{1}},\ldots ,p_{\sum_{n}}\}$ weighted with $w_{i}\in \Delta_{n}$ amounts to the symmetrized log-det Bregman centroid for the corresponding weighted set of positive-definite precision matrices $P_{\theta }=\left\{P_{1}=\sum_{1}^{-1},\ldots ,P_{n}=\sum_{n}^{-1}\right\}$. The symmetrized log-det Bregman barycenter C is the Riemannian geodesic midpoint A#H of the arithmetic barycenter $A=\sum_{i=1}^{n}w_{i}P_{i}$ and harmonic barycenter $H={\left(\sum_{i=1}^{n}w_{i}P_{i}^{-1}\right)}^{-1}$ where $X\#Y:=X^{\frac{1}{2}}\;{\left(X^{-\frac{1}{2}}\;Y\;X^{-\frac{1}{2}}\right)}^{\frac{1}{2}}\;X^{\frac{1}{2}}$ is the matrix geometric mean [40] G(X,Y)=X#Y:*

(5) $$C=\left(\sum_{i=1}^{n}w_{i}P_{i}\right)\#{\left(\sum_{i=1}^{n}w_{i}P_{i}^{-1}\right)}^{-1}.$$

Since the proof of this result mentioned in [29] was omitted in [29], we report a proof involving matrix analysis in full detail in Appendix B.

Next, we shall define two types of centers for sets of densities of a prescribed exponential family based on two different interpretations of Equation (5). We call them centers and not centroids because those points are defined by a generic structural formula instead of solutions of minimization problems of average divergences of Equation (1).

### 2.3. The Jeffreys–Fisher–Rao Center

Since an exponential family $E=\{p_{\theta }\left(x\right)\}$ induces the Riemannian manifold (M,g) with the Fisher metric *g* expressed in the θ-parameterization by the Fisher information matrix $\nabla^{2}F\left(\theta \right)$ and Fisher–Rao geodesics γ(p,q,t) defined with respect to the Levi-Civita connection $\bar{\nabla }$ (induced by *g*), we shall define the Jeffreys–Fisher–Rao center on M using the Fisher–Rao geodesics as follows:

**Definition** **2** (Jeffreys–Fisher–Rao (JFR) center)**.** *The Jeffreys–Fisher–Rao center $\theta_{\mathrm{JFR}}$ of a set $\left\{p_{\theta_{1}},\ldots ,p_{\theta_{n}}\right\}$ of weighted densities by $w\in \Delta_{n}$ is defined as the Fisher–Rao midpoint of the sided Kullback–Leibler centroids $\bar{\theta }=\sum_{i}w_{i}\theta_{i}$ and $\underline{\theta }={\left(\nabla F\right)}^{-1}\left(\sum_{i}w_{i}\nabla F\left(\theta_{i}\right)\right)$:*

(6) $$\theta_{\mathrm{JFR}}=\bar{\theta }\#\underline{\theta },$$

*where $p\#q=\gamma \left(p,q,\frac{1}{2}\right)$.*

Equation (6) is a generalization of Equation (5); therefore, the JFR center matches the Jeffreys centroid for same-mean multivariate normal distributions (Theorem 2).

Let $P_{\theta }=\left\{\theta_{1},\ldots ,\theta_{n}\right\}$ and $P_{\eta }=\left\{\eta_{1},\ldots ,\eta_{n}\right\}$, where η=∇F(θ) and $\theta =\nabla F^{*}\left(\eta \right)$. Denote by ${\mathrm{JFR}}_{F}\left(P_{\theta };w\right)$ the JFR center of θ-coordinate $\theta_{\mathrm{JFR}}$. Then, ${\mathrm{JFR}}_{F^{*}}\left(P_{\eta };w\right)$ = $\nabla F\left(\theta_{\mathrm{JFR}}\right):=\eta_{\mathrm{JFR}}$.

### 2.4. Gauss–Bregman Inductive Center

Another remarkable property of the Jeffreys centroid for a set $\left\{p_{\mu ,\sum_{1}},\ldots ,p_{\mu ,\sum_{n}}\right\}$ of same-mean multivariate normal distributions weighted by $w\in \Delta_{n}$ with arithmetic and harmonic means $A=\sum_{i=1}^{n}w_{i}\sum_{i}^{-1}$ and $H={\left(\sum_{i=1}^{n}w_{i}\sum_{i}\right)}^{-1}$ on the precision matrices $\sum_{1}^{-1},\ldots ,\sum_{n}^{-1}$, respectively, is that we have the following invariance of the Jeffreys centroid (see Lemma 17.4.4 of [29]):

(7) $$G\left(A,H\right)=G\left(\frac{A+H}{2},2\;{\left(A^{-1}+H^{-1}\right)}^{-1}\right).$$

Nakamura [41] defined the following double sequence scheme converging to the matrix geometry mean G(P,Q) for any two symmetric positive-definite matrices *P* and *Q*:

$$\begin{array}{ccc} P_{t+1} & = & A\left(P_{t},Q_{t}\right):=\frac{P_{t}+Q_{t}}{2},\\ Q_{t+1} & = & H\left(P_{t},Q_{t}\right):=2\;{\left(P_{t}^{-1}+Q_{t}^{-1}\right)}^{-1}, \end{array}$$

initialized with $P_{0}=P$ and $Q_{0}=Q$. We have $\lim_{t\to \infty }P_{t}=\lim_{t\to \infty }Q_{t}=P\#Q=G\left(P,Q\right)$. Let $P_{\infty }=\lim_{t\to \infty }P_{t}$ and $Q_{\infty }=\lim_{t\to \infty }Q_{t}$. That is, the geometric matrix mean can be obtained as the limits of a double sequence of means. We can thus approximate G(P,Q) by stopping the double sequence after *T* iterations to obtain

$$G^{\left(T\right)}\left(P,Q\right)=A\left(P_{T},Q_{T}\right)\approx G\left(P,Q\right).$$

Notice that we can recover those iterations from the invariance property of Equation (7): Indeed, we have

(8) $$G\left(P_{0},Q_{0}\right)=G\left(\underset{=:P_{1}}{\underset{︸}{A(P_{0},Q_{0})}},\underset{=:Q_{1}}{\underset{︸}{H(P_{0},Q_{0})}}\right)=G\left(\underset{=:P_{2}}{\underset{︸}{A(P_{1},Q_{1})}},\underset{=:Q_{2}}{\underset{︸}{H(P_{1},Q_{1})}}\right)=\ldots ,$$

and $∥P_{t}-Q_{t}∥=\sqrt{\mathrm{tr}(\left(P_{t}-Q_{t}\right)\left(P_{t}-Q_{t}\right))}$ decreases [41] as the number of iterations *t* increases. Thus, by induction, $G\left(P_{0},Q_{0}\right)=G\left(P_{\infty },Q_{\infty }\right)$ with $P_{\infty }=Q_{\infty }$. Since G(X,X)=X (means are reflexive), it follows that $G\left(P_{0},Q_{0}\right)=P_{\infty }=Q_{\infty }$. It is proved in [41] that the convergence rate of the sequence of double iterations is quadratic. This type of mean has been called an inductive mean [30,42] (or compound mean [43]) and originated from the Gauss arithmetic–geometric mean [44].

Our second interpretation of the geometric matrix mean of Equation (5) is to consider it as an inductive mean [30] and to generalize this double sequence process to pairs/sets of densities of an exponential family as follows:

**Definition** **3** (Gauss–Bregman (A,∇F) center)**.** *Let $P=\{p_{\theta_{1}},\ldots ,p_{\theta_{n}}\}$ be a set of n distributions of an exponential family with the cumulant function F(θ) weighted by a vector $w\in \Delta_{n}$. Then, the Gauss–Bregman inductive center $\theta_{\mathrm{GB}}$ is defined as the limit of the double sequence:*

$$\begin{array}{ccc} {\bar{\theta }}_{t+1} & = & A\left({\bar{\theta }}_{t},{\underline{\theta }}_{t}\right):=\frac{{\bar{\theta }}_{t}+{\underline{\theta }}_{t}}{2},\\ {\underline{\theta }}_{t+1} & = & m_{\nabla F}\left({\bar{\theta }}_{t},{\underline{\theta }}_{t}\right):={\left(\nabla F\right)}^{-1}\left(\frac{\nabla F\left({\bar{\theta }}_{t}\right)+\nabla F\left({\underline{\theta }}_{t}\right)}{2}\right), \end{array}$$

*initialized with ${\bar{\theta }}_{0}=\bar{\theta }=\sum_{i=1}^{n}w_{i}\theta_{i}$ (right Bregman centroid) and ${\underline{\theta }}_{0}=\underline{\theta }=\nabla F^{-1}\left(\sum_{i=1}^{n}w_{i}\nabla F\left(\theta_{i}\right)\right)$ (left Bregman centroid). That is, we have*

(9) $$\theta_{\mathrm{GB}}=\lim_{t\to \infty }{\bar{\theta }}_{t}=\lim_{t\to \infty }{\underline{\theta }}_{t}.$$

Let $\theta_{\mathrm{GB}}={\mathrm{GB}}_{F}\left(\bar{\theta },\underline{\theta }\right)$. Then, we have $\eta_{\mathrm{GB}}={\mathrm{GB}}_{F^{*}}\left(\bar{\eta },\bar{\theta }\right)=\nabla F\left(\theta_{\mathrm{GB}}\right)$. The Gauss–Bregman center $c_{\mathrm{GB}}$ has θ-coordinates $\theta_{G}B$ and η-coordinates $\eta_{\mathrm{GB}}$.

Algorithm 1 describes the approximation of the Gauss–Bregman inductive center by stopping the double sequence when the iterated centers are close enough to each other. We shall prove the matching convergence of those ${\bar{\theta }}_{t}$ and ${\underline{\theta }}_{t}$ sequences under separability conditions in Section 2.4.
**Algorithm 1:** Gauss–Bregman inductive center. 
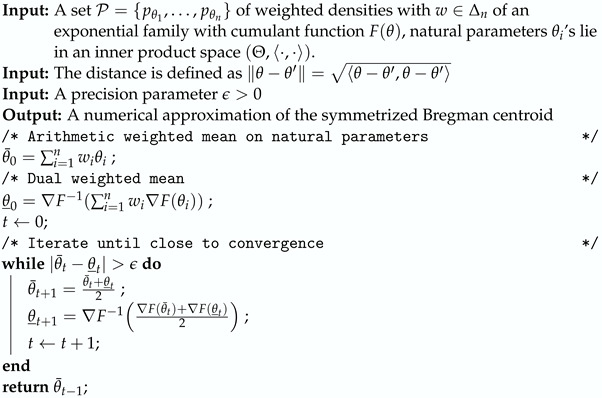


For example, the Gauss–Bregman center of two categorical distributions $p=(p_{1},\ldots ,p_{d})$ and $p^{\prime }=\left(p_{1}^{\prime },\ldots ,p_{d}^{\prime }\right)$ on a sample space X of *d* elements is obtained for the cumulant function $F\left(\theta \right)=\log\left(1+\sum_{i=1}^{d-1}e^{\theta_{i}}\right)$ with gradient $\nabla F\left(\theta \right)={\left[\eta_{i}=\frac{e^{\theta_{i}}}{1+\sum_{j=1}^{d-1}e^{\theta_{j}}}\right]}_{i}$ where $\theta =(\theta_{1}=\log\frac{p_{1}}{p_{d}},\ldots ,\theta_{d-1}=\log\frac{p_{d-1}}{p_{d}})$ is the natural parameter. The reciprocal gradient is ${\left(\nabla F\right)}^{-1}\left(\eta \right)={\left[\log\frac{\eta_{i}}{1-\sum_{j=1}^{d-1}\eta_{j}}\right]}_{i}$.

We may also compute the Gauss–Bregman center of two categorical distributions Cat(p) and $\mathrm{Cat}(p^{\prime })$ using iterations of arithmetic means $a_{t}$ and geometric normalized means $g_{t}$:

$$\begin{array}{ccc} a_{t+1}^{i} & = & A\left(a_{t}^{i},g_{t}^{i}\right):=\frac{a_{t}^{i}+g_{t}^{i}}{2},\;\forall i\in \left\{1,\ldots ,d\right\}\\ u_{t+1}^{i} & = & \sqrt{a_{t}^{i}g_{t}^{i}},\;\forall i\in \left\{1,\ldots ,d\right\},\\ g_{t+1}^{i} & = & \frac{u_{t+1}^{i}}{\sum_{j=1}^{d}u_{t+1}^{j}},\;\forall i\in \left\{1,\ldots ,d\right\}, \end{array}$$

where the $u_{t}$s are unnormalized geometric means and the $g_{t}$ represents normalized geometric means. We initialize the sequence with $a_{0}=p$ and $g_{0}=p^{\prime }$, and the Gauss–Bregman center is obtained in the limit $m_{\mathrm{GB}}^{\mathrm{Cat}}\left(p,p^{\prime }\right)=\lim_{t\to \infty }a_{t}=\lim_{t\to \infty }g_{t}$. See Algorithm 2.

The Jeffreys centroid of a set of centered multivariate normal distributions is the Gauss–Bregman center obtained for the generator $F\left(\theta \right)=-\frac{1}{2}\log\det\left(\theta \right)$, the cumulant function of the exponential family of centered normal distributions.
**Algorithm 2:** Gauss–Bregman inductive center for sets of categorical distributions. 
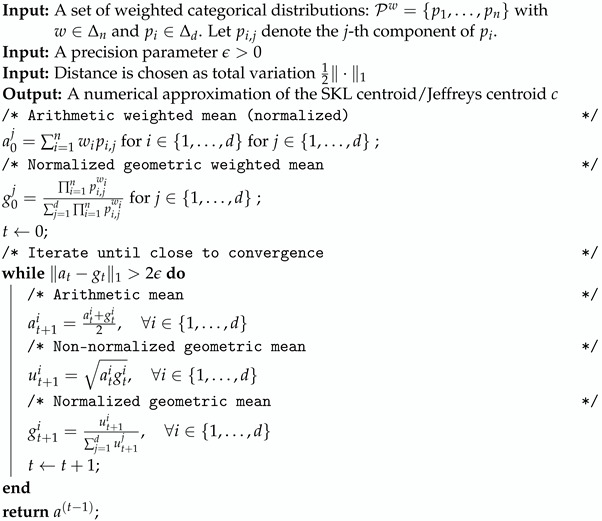


**Figure 2:**
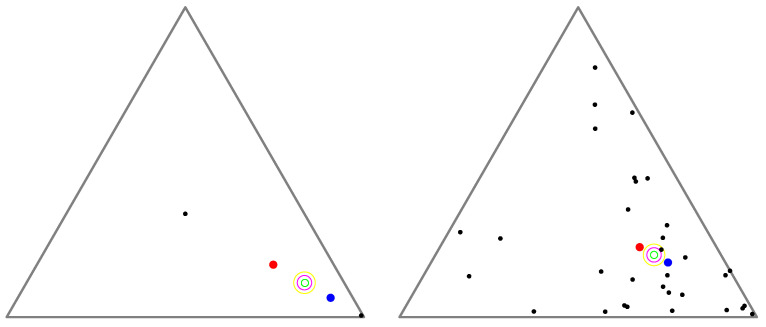
Visualizing the arithmetic and normalized geometric and numerical Jeffreys, Jeffreys–Fisher–Rao, and Gauss–Bregman centroids/centers in red, blue, green, purple, and yellow, respectively. (**Left**): input set consists of n=2 trinomial distributions (black) with parameters chosen randomly. (**Right**): input set consists of n=32 trinomial distributions (black) with parameters $\left(\frac{1}{2},\frac{1}{2}\right)$ and (0.99,0.005,0.005). The numerical Jeffreys centroid (green) is time consuming to calculate using the Lambert *W* function. However, the Jeffreys centroid can be well approximated by either the Jeffreys–Fisher–Rao center (purple) or the inductive Gauss–Bregman center (yellow). Point centers are visualized with different radii in order to distinguish them easily.

**Figure 3:**
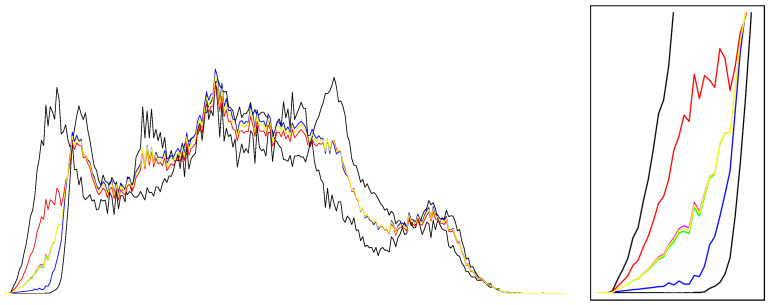
(**Left**): Displaying the arithmetic and normalized geometric and numerical Jeffreys, Jeffreys–Fisher–Rao, and Gauss–Bregman centroids/centers in red, blue, green, purple, and yellow, respectively. Input sets are two normalized histograms with d=256 bins plotted as polylines with 255 line segments (black). Observe that the Jeffreys–Fisher–Rao center (purple) and Gauss–Bregman center (yellow) approximates the Jeffreys centroid (green) well, which is more computationally expensive to calculate. (**Right**): close-up window on the first left bins of normalized histograms.

Figure 2 displays the arithmetic and normalized geometric and numerical Jeffreys, Jeffreys–Fisher–Rao, and Gauss–Bregman centroids/centers for a set of 32 trinomial distributions. We may consider normalized intensity histograms of images (modeled as multinomials with one trial) quantized with d=256 bins; that is, a normalized histogram with *d* bins is interpreted as a point in $\Delta_{d}$ and visualized as a polyline with d−1 line segments. Figure 3 (left) displays the various centroids and centers obtained for an input set consisting of two histograms (the commonly used Barbara and Lena images, which have been used in [31]). Notice that the JFR center (purple) and GB center (yellow) are close to the numerical Jeffreys centroid (green). We also provide a close-up window in Figure 3 (right).

Notice that we can experimentally check the quality of the approximation of the Gauss–Bregman center to the Jeffreys centroid by defining the symmetrized Bregman centroid energy:

$$E_{F}\left(\theta \right):=\langle \theta -\bar{\theta },\nabla F\left(\theta \right)\rangle -\langle \theta ,\nabla F\left(\bar{\theta }\right)\rangle ,$$

and checking that $\nabla E_{F}\left(\theta \right)$:

(10) $$\begin{array}{ccc} \forall i, & & \partial_{i}\left(\sum_{i=1}^{d}\left(\theta_{i}-{\bar{\theta }}_{i}\right)\partial_{i}F\left(\theta \right)-\theta_{i}\partial_{i}F\left(\bar{\theta }\right)\right)=0, \end{array}$$

(11) $$\begin{array}{ccc} & & \partial_{i}F\left(\theta \right)+\left(\theta_{i}-{\bar{\theta }}_{i}\right)\partial_{i}^{2}F\left(\theta \right)-\partial_{i}F\left(\bar{\theta }\right)+\left(\sum_{j\neq i}\left(\theta_{j}-{\bar{\theta }}_{j}\right)\partial_{i}\partial_{j}F\left(\theta \right)-\partial_{i}\theta_{j}\partial_{j}F\left(\bar{\theta }\right)\right)=0 \end{array}$$

is close to zero, where $\partial_{l}:=\frac{\partial }{\partial \theta_{l}}$.

Next, we study these two new types of centers and how well they approximate the Jeffreys centroid.

## 3. Gauss–Bregman Inductive Centers: Convergence Analysis and Properties

Let F(θ) be a strictly convex and differentiable real-valued function of Legendre type [45] defined on an open parameter space Θ. Then, the gradient map θ↦η(θ)=∇F(θ) is a bijection with the reciprocal inverse function $\eta \mapsto \theta \left(\eta \right)=\nabla F^{*}\left(\eta \right)={\left(\nabla F\right)}^{-1}\left(\eta \right)$ where $F^{*}\left(\eta \right)=\langle \eta ,\nabla F^{-1}\left(\eta \right)\rangle -F\left(\nabla F^{-1}\left(\eta \right)\right)$ is the Legendre–Fenchel convex conjugate. For example, we may consider the cumulant functions of regular exponential families.

We define the Gauss–Bregman center $\theta_{\mathrm{GB}}$ of a set $\left\{\theta_{1},\ldots ,\theta_{n}\right\}$ weighted by $w\in \Delta_{n}$ as the limits of the sequences ${\bar{\theta }}_{1},\ldots$ and ${\underline{\theta }}_{1},\ldots$ defined by

(12) $$\begin{array}{ccc} {\bar{\theta }}_{t+1} & = & A\left({\bar{\theta }}_{t},{\underline{\theta }}_{t}\right):=\frac{{\bar{\theta }}_{t}+{\underline{\theta }}_{t}}{2}, \end{array}$$

(13) $$\begin{array}{ccc} {\underline{\theta }}_{t+1} & = & m_{\nabla F}\left({\bar{\theta }}_{t},{\underline{\theta }}_{t}\right):={\left(\nabla F\right)}^{-1}\left(\frac{\nabla F\left({\bar{\theta }}_{t}\right)+\nabla F\left({\underline{\theta }}_{t}\right)}{2}\right), \end{array}$$

initialized with ${\bar{\theta }}_{0}=\bar{\theta }=\sum_{i=1}^{n}w_{i}\theta_{i}$ and ${\underline{\theta }}_{0}=\underline{\theta }=\nabla F^{-1}\left(\sum_{i=1}^{n}w_{i}\nabla F\left(\theta_{i}\right)\right)$. That is, we have

$$\theta_{\mathrm{GB}}=\lim_{n\to \infty }{\bar{\theta }}_{t}=\lim_{n\to \infty }{\underline{\theta }}_{t}.$$

**Figure 4:**
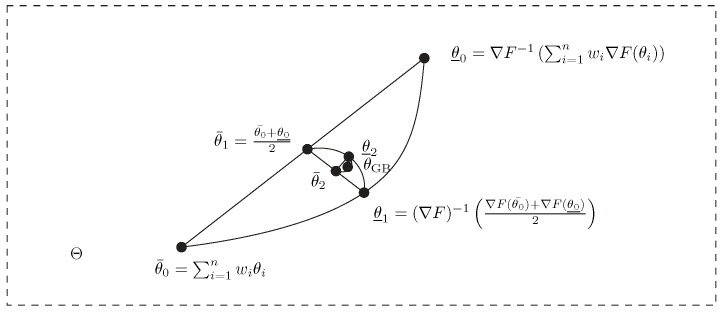
Geometric illustration of the double sequence inducing a Gauss–Bregman center in the limit.

Such a center has been called an inductive mean by Sturm [30]. See [42] for an overview of inductive means. Figure 4 geometrically illustrates the double sequence iterations converging to the Gauss–Bregman mean.

**Theorem** **3.** *The Gauss–Bregman (A,∇F) center with respect to a Legendre type function F(θ) is well defined (i.e., the double sequence converges) for separable Bregman generators.*

**Proof.**  We need to prove the convergence of $\left\{{\bar{\theta }}_{t}\right\}$ and $\left\{{\underline{\theta }}_{t}\right\}$ to the same finite limit. When F(θ) is univariate, the convergence of the inductive centers was reported in [43]. We need to prove that the double iterations of Equations (12) and (13) converge.

**Figure 5:**
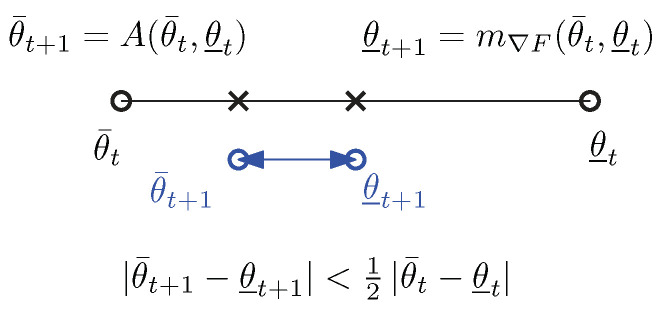
Illustration of the double sequence convergence for scalar Gauss–Bregman $\left(A,m_{\nabla F}\right)$ mean.

Let us consider the following cases:

1. When the dimension is one, the quasi-arithmetic mean $m_{f^{\prime }}$ for *f*, a strictly convex and differentiable function, lies between the minimal and maximal argument (i.e., this is the definition of a strict mean):
  $$\min\left\{\theta_{1},\theta_{2}\right\}\leq m_{f^{\prime }}\left(\theta_{1},\theta_{2}\right)\leq \max\left\{\theta_{1},\theta_{2}\right\}.$$
  Thus, we have
  $$|{\bar{\theta }}_{t+1}-{\underline{\theta }}_{t+1}|\leq \frac{1}{2}\left|{\bar{\theta }}_{t}-{\underline{\theta }}_{t}\right|,$$
  and it follows that $|{\bar{\theta }}_{t+1}-{\underline{\theta }}_{t+1}|\leq \frac{1}{2^{t}}\left|{\bar{\theta }}_{0}-{\underline{\theta }}_{0}\right|$. Thus, we have quadratic convergence of scalar $\left(A,f^{\prime }\right)$ means. See Figure 5.
2. When F(θ) is multivariate and separable, i.e., $F\left(\theta \right)=\sum_{i=1}^{d}f_{i}\left(\theta^{i}\right)$ where $\theta =(\theta^{1},\ldots ,\theta^{d})$ are the components of $\theta \in R^{d}$ and the $f_{i}$s are scalar strictly convex and differentiable functions, we can apply case 1 dimension-wise to obtain the quadratic convergence.
3. Otherwise, we consider the multivariate quasi-arithmetic center $m_{\nabla F}\left(\theta ,\theta^{\prime }\right)$ with the uniform weight vector $w=(\frac{1}{2},\frac{1}{2})$. One problem we face is that the quasi-arithmetic center $m_{\nabla F}\left(\theta ,\theta^{\prime }\right)$ for $\theta \neq \theta^{\prime }$ may lie outside the open bounding box of $R^{d}$ with diagonal corners θ and $\theta^{\prime }$:
  $$\theta_{m}=\left(\min\left\{\theta^{1},{\theta^{\prime }}^{1}\right\},\ldots ,\min\left\{\theta^{d},{\theta^{\prime }}^{d}\right\}\right),\;\theta_{M}=\left(\max\left\{\theta^{1},{\theta^{\prime }}^{1}\right\},\ldots ,\max\left\{\theta^{d},{\theta^{\prime }}^{d}\right\}\right).$$
  Indeed, in the 2D case, we may consider θ=(x,y) and $\theta^{\prime }=\left(x^{\prime },y\right)$. Clearly, the open bounding box is empty, and the midpoint $m_{\nabla F}\left(\theta ,\theta^{\prime }\right)$ lies outside this box. Yet, we are interested in the convergence rate when $\theta^{\prime }\approx \theta$.
  In general, we shall measure the difference between two iterations by the squared norm distance induced by the inner product:
  $$∥A\left(\theta ,\theta^{\prime }\right)-m_{\nabla F}\left(\theta ,\theta^{\prime }\right){∥}^{2}=\langle A\left(\theta ,\theta^{\prime }\right)-m_{\nabla F}\left(\theta ,\theta^{\prime }\right),A\left(\theta ,\theta^{\prime }\right)-m_{\nabla F}\left(\theta ,\theta^{\prime }\right)\rangle .$$

 □

Let $m_{F}^{\mathrm{GB}}\left(\theta_{1},\theta_{2}\right)$ denote the Gauss–Bregman center of $\theta_{1}$ and $\theta_{2}$, $A\left(\theta_{1},\theta_{2}\right)=\frac{\theta_{1}+\theta_{2}}{2}$ the arithmetic mean, and $m_{\nabla F}\left(\theta_{1},\theta_{2}\right)={\left(\nabla F\right)}^{-1}\left(\frac{\nabla F\left(\theta_{1}\right)+\nabla F\left(\theta_{2}\right)}{2}\right)$ the quasi-arithmetic center.

By construction, the Gauss–Bregman center enjoys the following invariance property generalizing Lemma 17.4.4 of [29] in the case of the log det generator:

**Property** **1.** *We have $m_{F}^{\mathrm{GB}}\left(\theta_{1},\theta_{2}\right)=m_{F}^{\mathrm{GB}}\left(A\left(\theta_{1},\theta_{2}\right),m_{\nabla F}\left(\theta_{1},\theta_{2}\right)\right)$.*

**Proof.**  Similar to the cascaded inequalities of Equation (8), we have

(14) $$m_{F}^{\mathrm{GB}}\left(\theta_{1},\theta_{2}\right)=m_{\mathrm{GB}}^{F}\left(\underset{=:\theta_{1}^{\left(1\right)}}{\underset{︸}{A(\theta_{1},\theta_{2})}},\underset{=\theta_{2}^{\left(1\right)}}{\underset{︸}{m_{\nabla F}\left(\theta_{1},\theta_{2}\right)}}\right)=\ldots$$

In the limit t→∞, we have $m_{F}^{\mathrm{GB}}\left(\theta_{1},\theta_{2}\right)=m_{F}^{\mathrm{GB}}\left(\theta_{1}^{\left(\infty \right)},\theta_{2}^{\left(\infty \right)}\right)=m_{F}^{\mathrm{GB}}\left(\theta_{1}^{\left(\infty -1\right)},\theta_{2}^{\left(\infty -1\right)}\right)=\ldots$ Since ∞−1=∞, we obtain the desired invariance property:

$$m_{F}^{\mathrm{GB}}\left(\theta_{1},\theta_{2}\right)=m_{F}^{\mathrm{GB}}\left(A\left(\theta_{1},\theta_{2}\right),m_{\nabla F}\left(\theta_{1},\theta_{2}\right)\right).$$

 □

Note that when F(θ) is univariate, the Gauss–Bregman mean $m_{F}^{\mathrm{GB}}\left(\theta_{1},\theta_{2}\right)$ converges at a quadratic rate [43]. In particular, when F(θ)=−logθ (Burg negentropy), we have $F^{\prime }\left(\theta \right)=-\frac{1}{\theta }$ ($m_{F^{\prime }}$ is the harmonic mean) and the Gauss–Bregman mean is the arithmetic–harmonic mean (AHM) which converges to the geometric mean, a simple closed-form formula. Notice that the geometric mean $g=\sqrt{xy}$ of two scalars x>0 and y>0 can be expressed using the arithmetic mean $a=\frac{x+y}{2}$ and the harmonic mean $h=\frac{2xy}{x+y}$: $g=\sqrt{ah}$. But when F(θ)=θlogθ−θ (Shannon negentropy), the Gauss–Bregman mean $m_{F}^{\mathrm{GB}}\left(\theta_{1},\theta_{2}\right)$ coincides with the Gauss arithmetic–geometric mean [44] (AGM) since $F^{\prime }\left(\theta \right)=\log\theta$ and $m_{F^{\prime }}\left(\theta_{1},\theta_{2}\right)=\sqrt{\theta_{1}\theta_{2}}$, the geometric mean. Thus, $m_{F}^{\mathrm{GB}}\left(\theta_{1},\theta_{2}\right)$ is related to the elliptic integral *K* of the first type [44]: there is no closed-form formula for the AGM in terms of elementary functions as this induced mean is related to the complete elliptic integral of the first kind K(·):

(15) $$\mathrm{AGM}\left(x,y\right)=\frac{\pi }{4}\frac{x+y}{K\left(\frac{x-y}{x+y}\right)},$$

where $K\left(u\right)=\int_{0}^{\frac{\pi }{2}}\frac{d\theta }{\sqrt{1-u^{2}\sin^{2}\left(\theta \right)}}$ is the elliptic integral. Thus, it is difficult, in general, to report a closed-form formula for the inductive Gauss–Bregman means even for univariate generators F(θ).

The Jeffreys centroid of x>0 and y>0 with respect to the scalar Jeffreys divergence $D_{J}\left(p,q\right)=\left(p-q\right)\log\frac{p}{q}$ admits a closed-form solution [31]:

(16) $$c=\frac{a}{W_{0}\left(\frac{a}{g}e\right)}$$

where $a=\frac{x+y}{2}$ and $g=\sqrt{xy}$ and $W_{0}$ is the principal branch of the Lambert *W* function [33]. This example shows that the Gauss–Bregman center does not coincide with the Jeffreys centroid in general (e.g., compare Equation (15) with Equation (16)).

## 4. Jeffreys–Fisher–Rao Centers: Generic Structural Formula and Some Closed-Form Formula

### 4.1. Jeffreys–Fisher–Rao Center for Uni-Parametric Statistical Models

Consider a set $P=\{p_{\theta_{1}},\ldots ,p_{\theta_{n}}\}$ of *n* parametric distributions where θ∈Θ⊂R is a scalar parameter. Let $w=\left(w_{1},\ldots ,w_{n}\right)\in \Delta_{n}$ be a weight vector on P such that the weight of $p_{\theta_{i}}$ is $w_{i}$. The distributions $p_{\theta }$s may not necessarily belong to an exponential family (e.g., the Cauchy scale family). The Fisher–Rao geometry [46,47] of the parametric family of distributions $F=\{p_{\theta }\;:\;\theta \in \Theta \}$ (the statistical model) can be modeled as a Riemannian manifold with the Fisher metric g(θ)=I(θ) defined by the Fisher information $I\left(\theta \right)=E_{\theta }\left[{\left(\log p_{\theta }\left(x\right)\right)}^{2}\right]=-E_{\theta }\left[\nabla^{2}\log p_{\theta }\left(x\right)\right]$. When F is an exponential family with the cumulant function F(θ), we have $I\left(\theta \right)=F^{\prime \prime }\left(\theta \right)$.

The underlying geometry of (F,g(θ)=I(θ)) is Euclidean after a change in variable $\eta \left(\theta \right)=\sqrt{I(\theta )}$ since we can write the metric tensor as follows:

$$g\left(\theta \right)=\sqrt{I(\theta )}\;\times \underset{=g_{\mathrm{Euclidean}}}{\underset{︸}{1}},\times \;\sqrt{I(\theta )}.$$

Thus, the Riemannian Fisher–Rao distance is the Euclidean distance expressed in the h(θ)-coordinate system with $h\left(\theta \right)=\int_{\theta_{0}}^{\theta }\sqrt{I(u)}\;du$, and we have the Fisher–Rao distance given by

$$\rho \left(p_{\theta_{1}},p_{\theta_{2}}\right)=\left|h\left(\theta_{1}\right)-h\left(\theta_{2}\right)\right|.$$

When F is an exponential family with the cumulant function f(θ), we have $I\left(u\right)=f^{\prime \prime }\left(u\right)$.

We summarize the result on the JFR center in the following theorem:

**Theorem** **4** (Jeffreys–Fisher–Rao centroid in uni-order exponential families)**.** *The Jeffreys–Fisher–Rao centroid $\theta_{S}$ of n densities $p_{\theta_{1}},\ldots ,p_{\theta_{n}}$ of an exponential family of order one with the log-normalizer f(θ) for θ∈Θ, the natural parameter space, and weight vector $w\in \Delta_{n}$ is*

(17) $$\theta_{S}=m_{h}\left(\bar{\theta },\underline{\theta }\right),$$

*where $m_{h}\left(\bar{\theta },\underline{\theta }\right)=h^{-1}\left(\frac{h\left(\bar{\theta }\right)+h\left(\underline{\theta }\right)}{2}\right)$ is the quasi-arithmetic mean [35] of the dual left and right KL centroids $\bar{\theta }=\sum_{i=1}^{n}w_{i}\theta_{i}=\theta_{R}$ and $\underline{\theta }={\left(f^{\prime }\right)}^{-1}\left(\sum_{i=1}^{n}w_{i}f^{\prime }\left(\theta_{i}\right)\right)$ with respect to the scalar monotone function $h=\int_{\theta_{0}}^{\theta }\sqrt{f^{\prime \prime }\left(u\right)}\;du$ for any θ∈Θ.*

**Proof.**  Since the Fisher information is $I\left(\theta \right)=f^{\prime \prime }\left(\theta \right)$, we have $h\left(\theta \right)=\int_{\theta_{0}}^{\theta }\sqrt{f^{\prime \prime }\left(u\right)}du$. The Riemannian center of mass [48] minimizes

$$\theta_{S}=\arg\min_{\theta }\sum_{i=1}^{n}w_{i}\rho^{2}\left(\theta_{i},\theta \right).$$

But in the *h*-parameterization, the Riemannian centroid, amounts to a Euclidean center of mass/centroid in the *h*-Cartesian coordinate system:

$$h\left(\theta_{S}\right)=\sum_{i=1}^{n}w_{i}h\left(\theta_{i}\right).$$

Therefore, we have $\theta_{S}=h^{-1}\left(\sum_{i}w_{i}h\left(\theta_{i}\right)\right)=:m_{h}\left(\theta_{1},\ldots ,\theta_{n};w_{1},\ldots ,w_{n}\right)$, a weighted quasi-arithmetic mean. Since the Jeffreys centroid amounts to a symmetrized Bregman centroid of the left and right Bregman centroids [28], $\underline{\theta }=m_{f^{\prime }}\left(\theta_{1},\ldots ,\theta_{n};w_{1},\ldots ,w_{n}\right)$ and $\bar{\theta }=\sum_{i}w_{i}\theta_{i}$. It follows that the Jeffreys–Fisher–Rao center is $\theta_{\mathrm{JFR}}=m_{h}\left(\bar{\theta },\underline{\theta }\right)$ after using Property 3. □

### 4.2. Jeffreys–Fisher–Rao Center for Categorical Distributions

Recall from Theorem 1 that the Jeffreys centroid $c=(c_{1},\ldots ,c_{j},\ldots ,c_{d})$ of a set of *n* categorical distributions with parameters arranged in the matrix $\left[p_{i,j}\right]$ is given by

$$c_{j}\left(\lambda \right)=\frac{a_{j}}{W_{0}\left(\frac{a_{j}}{g_{j}}\;e^{1+\lambda }\right)},\;\forall j\in \left\{1,\ldots ,d\right\},$$

where $a_{j}=\sum_{i=1}^{n}w_{i}p_{i,j}$ and $g_{j}=\frac{\prod_{i=1}^{n}p_{i,j}^{w_{i}}}{\sum_{j=1}^{d}\prod_{i=1}^{n}p_{i,j}^{w_{i}}}$ are the components of the weighted arithmetic and normalized geometric means, respectively, and $W_{0}$ is the principal branch of the Lambert *W* function [33]. The optimal λ≤0 is unique and satisfies $\lambda =-D_{\mathrm{KL}}\left(c_{j}\left(\lambda \right):g\right)$.

Let $c\left(\lambda \right)=(c_{1}\left(\lambda \right),\ldots ,c_{d}\left(\lambda \right))$. Let $L_{J}\left(p\right)$ denote the Jeffreys loss function to minimize to find the optimal Jeffreys centroid:

(18) $$L_{J}\left(p\right)=\sum_{i=1}^{n}w_{i}D_{J}\left(p_{i},p\right)$$

We say that *p* is a (1+ϵ) approximation of the exact Jeffreys centroid *c* when we have

$$L_{J}\left(c\right)\leq L_{J}\left(p\right)\leq \left(1+\epsilon \right)L_{J}\left(c\right).$$

It was shown in [31] that $\tilde{c}=c\left(0\right)$, called the unnormalized Jeffreys center, yields a s(λ)−1 approximation on *c* where $s\left(\lambda \right)=\sum_{j}c_{j}\left(\lambda \right)\leq 1$.

Since the Fisher–Rao geodesic midpoints on the categorical Fisher–Rao manifold are known in closed form [49], we give the mathematical expression of the JFR center as follows:

**Theorem** **5** (JFR centroid of categorical distributions)**.** *Let $P_{w}=\left\{p_{1},\ldots ,p_{n}\right\}$ be a set of n probability mass functions weighted by $w\in \Delta_{n}$ with $p_{i}=\left(p_{i,1},\ldots ,p_{i,d}\right)\in \Delta_{d}$ for i∈{1,…,n} and $w\in \Delta_{n}$. Then, the JFR barycenter c minimizing is unique and given by the following formula:*

(19) $$c_{j}=\frac{{\left(\sqrt{a_{j}}+\sqrt{g_{j}}\right)}^{2}}{2\;(1+\sum_{l=1}^{d}\sqrt{a_{j}}\sqrt{g_{j}})},\forall j\in \left\{1,\ldots ,d\right\},$$

*where $a=\left(a_{1},\ldots ,a_{d}\right)=\sum_{i=1}^{n}w_{i}p_{i}$ is the weighted arithmetic mean and $g=(g_{1},\ldots ,g_{d})$ is the normalized weighted geometric mean with components $g_{j}=\frac{\prod_{i=1}^{n}p_{i,j}^{w_{i}}}{\sum_{j=1}^{d}\prod_{i=1}^{n}p_{i,j}^{w_{i}}}$ for i∈{1,…,d}.*

Notice that the JFR center differs from the Jeffreys centroid, which requires the use of the Lambert *W* function [33]. However, we noticed that for practical applications, the JFR centroid approximates the Jeffreys centroid well and is much faster to compute (see the experiments in Section 5).

### 4.3. Jeffreys–Fisher–Rao Center for Multivariate Normal Distributions

Let $P=\{p_{\mu_{1},\sum_{1}},\ldots ,p_{\mu_{n},\sum_{n}}\}$ be a set of *n* probability density functions (PDFs) of *d*-variate normal distributions weighted by $w\in \Delta_{n}$, where the PDF of a multivariate normal distribution of mean μ and the covariance matrix ∑ is given by

$$p_{\mu ,\sum }=\frac{1}{{\left(2\pi \right)}^{\frac{d}{2}}\sqrt{\det(\sum )}}\;\exp\left(-\frac{1}{2}{\left(x-\mu \right)}^{⊤}\sum^{-1}\left(x-\mu \right)\right).$$

Let $\lambda_{i}=\left(\mu_{i},\sum_{i}\right)$ be the ordinary parameterization of normal distributions $p_{\mu_{i},\sum_{i}}$. The family $F=\{p_{\mu ,\sum }\left(x\right)\;:\;\mu \in R^{d},\sum \in {\mathrm{Sym}}^{++}\left(R,d\right)\}$ of multivariate normal distributions forms an exponential family with the dual natural θ- and moment η-parameterizations [7] given by

$$\begin{array}{ccc} \theta (\lambda ) & = & \left(\theta_{v},\theta_{M}\right)=\left(\sum^{-1}\mu ,\frac{1}{2}\sum^{-1}\right),\\ \eta (\lambda ) & = & \left(\mu ,\mu \mu^{⊤}+\sum \right), \end{array}$$

when choosing the sufficient statistic $t\left(x\right)=\left(x,xx^{⊤}\right)$. The Jeffreys divergence between two *d*-variate normal distributions $N(\mu_{1},\sum_{1})$ and $N(\mu_{2},\sum_{2})$ is given by the formula

$$D_{J}\left(p_{\mu_{1},\sum_{1}},p_{\mu_{2},\sum_{2}}\right)=\left(\mu_{2}-\mu_{1}\right)⊤\left(\sum_{1}^{-1}+\sum_{2}^{-1}\right)\left(\mu_{2}-\mu_{1}\right)+\mathrm{tr}\left(\sum_{1}^{-1}\sum_{2}+\sum_{2}^{-1}\sum_{1}\right)-2d.$$

The left and right Kullback–Leibler barycenters amount to the corresponding right and left Bregman barycenters [28] induced by the cumulant function

$$F\left(\theta \right)=F\left(\theta_{v},\theta_{M}\right)=\frac{1}{2}\left(d\log\pi -\log\det\left(\theta_{M}\right)+\frac{1}{2}\theta_{v}^{⊤}\theta_{M}^{-1}\theta_{v}\right),$$

and the gradient of F(θ) defines the dual moment parameter with

$$\eta \left(\theta \right)=\nabla F\left(\theta \right)=\left(\frac{1}{2}\theta_{M}^{-1}\theta_{v},\frac{1}{2}\theta_{M}^{-1}-\frac{1}{4}\left(\theta_{M}^{-1}\theta_{v}\right){\left(\theta_{M}^{-1}\theta_{v}\right)}^{⊤}\right).$$

The reciprocal gradient is given by

$$\theta \left(\eta \right)=\theta \left(\eta_{v},\eta_{M}\right)={\left(\nabla F\right)}^{-1}\left(\eta \right)=\left(\theta_{v}=-{\left(\eta_{M}+\eta_{v}\eta_{v}^{⊤}\right)}^{-1}\eta_{v},\theta_{M}=-\frac{1}{2}{\left(\eta_{M}+\eta_{v}\eta_{v}^{⊤}\right)}^{-1}\right).$$

The Gauss–Bregman center is a $\left(A,m_{\nabla F}\right)$-inductive center, which can be approximated by carrying a prescribed number *T* of iterations of the Gauss–Bregman double sequence.

Although the Rao distance between two *d*-variate normal distributions is not available in closed form when d>1 [50,51], the Jeffreys–Fisher–Rao center can be computed in closed form. Indeed, the sided Kullback–Leibler centroids of multivariate normal distributions amount to reverse-sided Bregman centroids [28], and the Fisher–Rao geodesic midpoint between two multivariate normal distributions was recently reported in [32]. Appendix C concisely describes the method of Kobayashi [32], which allows one to obtain the Fisher–Rao midpoints of multivariate normal distributions. An implementation of that algorithm is available in the Python software library pyBregMan [52].

Thus, the Jeffreys–Fisher–Rao center is available in closed form:

**Theorem** **6** (JFR center of MVNs)**.** *The Jeffreys–Fisher–Rao center of a finite set of weighted multivariate normal distributions is available in closed form.*

**Figure 6:**
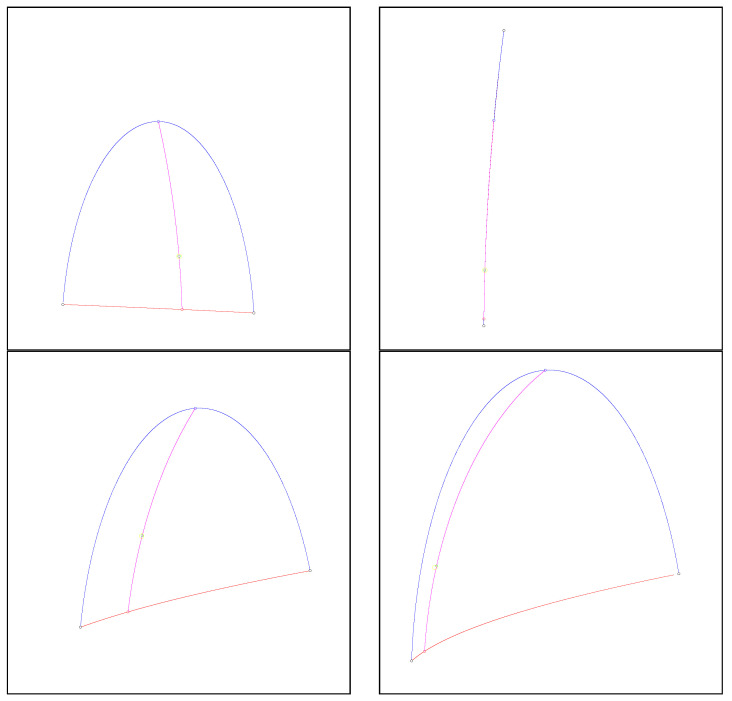
Visualization of the Jeffreys–Fisher–Rao center and Gauss–Bregman center of two univariate normal distributions (black circle). The exponential geodesic and mixture geodesics are shown in red and blue, respectively, with their corresponding midpoints. The Jeffreys–Fisher–Rao is the Fisher–Rao midpoint (green) lying on the Fisher–Rao geodesics (purple). The inductive Gauss–Bregman center is displayed in yellow with double size in order to ease its comparison with the Jeffreys–Fisher–Rao center.

Note that the Fisher–Rao distance between normal distributions is invariant under the action of the positive affine group [50], as are the Jeffreys centroid, the JFR center, and the GB center. Figure 6 shows several examples of the JFR and GB centers of two univariate normal distributions. We can observe that those centers are close to each other although they are distinct when the normal distributions do not share the same means and covariance matrices.

**Figure 7:**
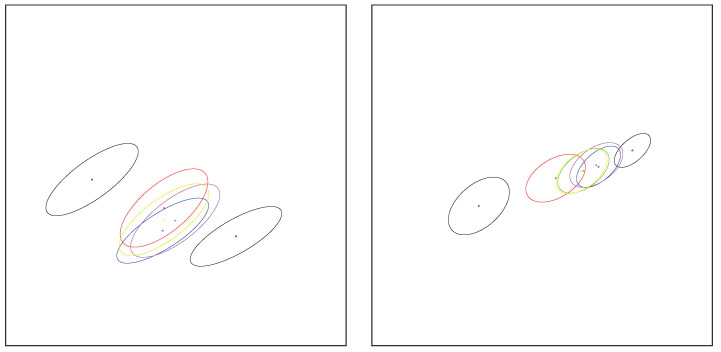
Centroids and centers between a pair of bivariate normal distributions (black). Each normal distribution N(μ,∑) (parameterized by a 5D parameter θ) is displayed as a 2D ellipsoid $E\left(\mu ,\sum \right)=\{{\left(x-\mu \right)}^{⊤}\sum^{-1}\left(x-\mu \right)=l\}$ for a prescribed level l>0 in the sample space $R^{2}$. Blue, red, purple, yellow, and green ellipsoids correspond to *m*-geodesic midpoint, *e*-geodesic midpoint, Jeffreys–Fisher–Rao midpoint, Gauss–Bregman inductive mean, and numerical Jeffreys centroid (symmetrized Bregman centroid), respectively.

Figure 7 shows the various centroids/centers between two bivariate normal distributions displayed as ellipsoids centered as their means. Observe that the inductive Gauss–Bregman center is visually closer than the Jeffreys–Fisher–Rao center to the Jeffreys centroid.

**Figure 8:**
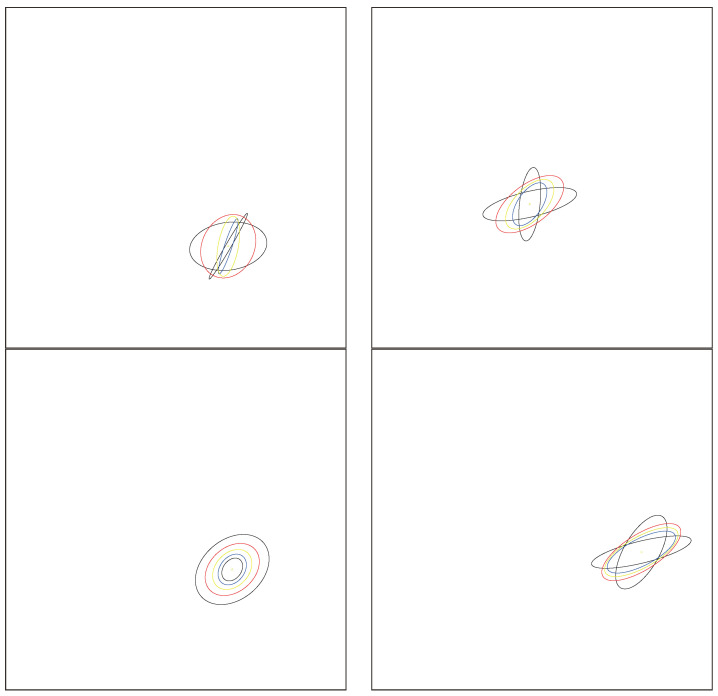
Centroids and centers between a pair of bivariate centered normal distributions (black). Each normal distribution N(μ,∑) with a prescribed μ (parameterized by a 3D parameter θ) is displayed as a 2D ellipsoid. The red and blue ellipsoids correspond to the *e*-geodesic and *m*-geodesic midpoints, respectively. The green ellipsoid is the exact Jeffreys centroid which coincide perfectly with the inductive Gauss–Bregman center (yellow) and Jeffreys–Fisher–Rao center (purple). Thus these three green, yellow, and purple matching ellipsoids are rendered superposed in an overall shade of brown.

**Figure 9:**
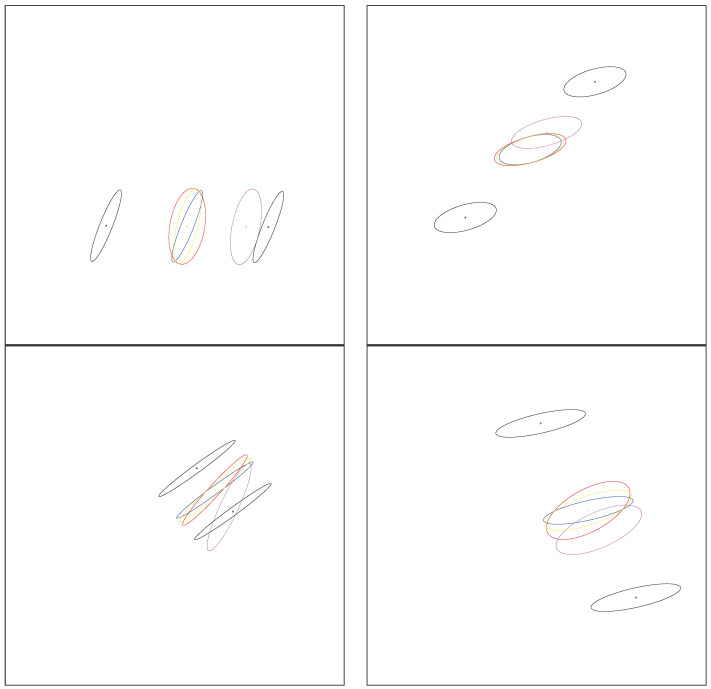
Centroids and centers between a pair of bivariate-centered normal distributions (black). Each normal distribution N(μ,∑) with a prescribed covariance matrix ∑ (parameterized by a 2D parameter θ) is displayed as a 2D ellipsoid. The red and blue ellipsoids correspond to the *e*-geodesic and *m*-geodesic midpoints, respectively. The inductive Gauss–Bregman (yellow) and Jeffreys–Fisher–Rao center (purple) do not coincide.

Figure 8 displays the various centroids and centers for pairs of bivariate normal distributions centered at the same mean. Figure 9 shows the centroids and centers for pairs of bivariate normal distributions with the same covariance matrix.

**Remark** **1.** *In general, an exponential family may be characterized equivalently by two convex functions: (1) its log-normalizer F(θ) or (2) its partition function Z(θ)=exp(F(θ)), which is log-convex and hence also convex [53]. It has been shown that the Bregman divergence $B_{Z}$ for $Z=\sqrt{\det(\theta )}$ (convex) corresponds to the reverse extended Kullback–Leibler divergence between unnormalized PDFs of normal distributions:*

$$B_{Z}\left(\theta_{1}:\theta_{2}\right)=D_{\mathrm{KL}}^{+}\left({\tilde{p}}_{\lambda (\theta_{2})}:{\tilde{p}}_{\lambda (\theta_{1})}\right),$$

*where ${\tilde{p}}_{\mu ,\sum }=\exp\left(-\frac{1}{2}{\left(x-\mu \right)}^{⊤}\sum^{-1}\left(x-\mu \right)\right)$ and the extended KLD between two positive measures is given by*

$$D_{\mathrm{KL}}^{+}\left(m_{1}:m_{2}\right)=\int \left(m_{1}\left(x\right)\log\frac{m_{1}\left(x\right)}{m_{2}\left(x\right)}+m_{2}\left(x\right)-m_{1}\left(x\right)\right)d\mu \left(x\right).$$

**Remark** **2.** *We may further define yet another center for multivariate normal distributions by considering the Fisher–Rao isometric embedding of the Fisher–Rao d-variate normal manifold $M=\{p_{\mu ,\sum }\}$ into the Fisher–Rao (d+1)-variate centered manifold $N_{0}^{+}=\left\{q_{P}\left(y\right)=p_{0,P}\left(y\right)\;:\;P\in {\mathrm{Sym}}^{++}\left(R,d+1\right)\right\}$ using Calvo and Oller mapping [50]:*

$$f\left(\mu ,\sum \right):=\left[\begin{array}{cc} \sum +\mu \mu^{⊤} & \mu \\ \mu^{⊤} & 1 \end{array}\right].$$

*Let $\bar{M}=\left\{f\left(p\right)\;:\;p\in M\right\}$ denote the embedded submanifold of codimension one in $N_{0}^{+}$. The Calvo–Oller center is then defined by taking the Fisher–Rao midpoints $q_{\mathrm{CO}}$ of $q_{P_{1}}$ and $q_{P_{2}}$, projecting $q_{\mathrm{CO}}$ onto $\bar{M}$ as $q_{\mathrm{CO}}^{\prime }$ and converting $q_{\mathrm{CO}}^{\prime }$ into $p_{\mathrm{CO}}\in M$ using the inverse mapping $f^{-1}$ [51].*

*The Fisher orthogonal projection of a (d+1)×(d+1) matrix $P\in N_{0}^{+}$ onto the submanifold $\bar{M}$ is performed as follows: Let $\beta =P_{d+1,d+1}$ and write $P=\left[\begin{array}{cc} \sum +\beta \mu \mu^{⊤} & \beta \mu \\ \beta \mu^{⊤} & \beta \end{array}\right]$. Then, the orthogonal projection at P∈P onto $\bar{M}$ is $\left[\begin{array}{cc} \sum +\mu \mu^{⊤} & \mu^{⊤}\\ \mu & 1 \end{array}\right]$. See [51] for details of the Calvo and Oller embedding/projection method.*

## 5. Experiments

We run all experiments on a Dell Inspiron 5502 Core i7-116567@2.8 GHz using compiled Java programs. For each experiment, we consider a set of n=2 uniformly randomized histograms with *d* bins (i.e., points in $\Delta_{d}$) and calculate the numerical Jeffreys centroid, which requires the time-consuming Lambert *W* function, the GB center, and the JFR center. For each prescribed value of *d*, we run 10,000 experiments to collect various statistics like the average and maximum approximations and running times. The approximations of the JFR and GB methods are calculated either as the approximation of the Jeffreys information (Equation (18)) or as the approximation of the centers with respect to the numerical Jeffreys centroids measured using the total variation distance. Table 1 is a verbatim export of our experimental results when we range the dimension of histograms for d=2 to d=256 by doubling the dimension at each round. The inductive GB center is stopped when the total variation $\frac{1}{2}{∥a_{t}-g_{t}∥}_{1}\leq 10^{-8}$.

We observe that the JFR center is faster to compute than the GB center but the GB center is of higher quality (i.e., a better approximation with a lower ϵ) than the JFR center to approximate the numerical Jeffreys centroid.

Another test consists of choosing d=3 and the following two 3D normalized histograms: $\left(\frac{1}{3},\frac{1}{3},\frac{1}{3}\right)$ and (1−α,α/2,α/2) for $\alpha \in \{10^{-1},10^{-2},\ldots ,10^{-7},10^{-8}\}$. Table 2 reports the experiments. The objective is to find a setting where both the JFR and GB centers are distinguished from the Jeffreys centroid. We see that as we decrease α, the approximation factor ϵ gets worse for both the JFR center and the GB center. The JFR center is often faster to compute than the GB inductive center, but the approximation of the GB center is better than the JFR approximation.

Finally, we implemented the Gauss–Bregman and Jeffreys–Fisher–Rao centers and Jeffreys centroid using multi-precision arithmetic. We report the following experiments using 200-digit precision arithmetic for the following input of two normalized histograms: p=(0.1,0.9) and q=(0.8,0.2). We report the various first 17-digit mantissas obtained with the corresponding Jeffreys information:

- Jeffreys center: (0.42490383904214813,0.575096160957851866)
  Jeffreys information: 1.2490723231955352.
- Gauss–Bregman center: (**0.424903839042**76856, **0.575096160957**231439)
  Jeffreys information: 1.2490723231955353.
- Jeffreys–Fisher–Rao center: (**0.42490390**202906282, **0.575096**097970937175)
  Jeffreys information: 1.2490723232068266.

The total variation distance between the Jeffreys centroid and the Gauss–Bregman center is $6.204271148284087422350000686372\;10^{-13}$.

The total variation distance between the Jeffreys centroid and the Jeffreys–Fisher–Rao center is $6.298691469047911984039363762611\;10^{-8}$.

The total variation distance between the Gauss–Bregman center and the Jeffreys–Fisher–Rao center is $6.298629426336429143165140262604\;10^{-8}$.

Although all those points are close to each other, they are all distinct points (note that using the limited precision of the IEEE 754 floating point standard may yield a misleading interpretation of experiments).

## 6. Conclusions and Discussion

In this work, we considered the Jeffreys centroid of a finite weighted set of densities of a given exponential family $E=\{p_{\theta }\left(x\right)\;:\;\theta \in \Theta \}$. This Jeffreys centroid amounts to a symmetrized Bregman centroid on the corresponding weighted set of natural parameters of the densities [28]. In general, the Jeffreys centroids do not admit closed-form formulas [28,31] except for sets of same-mean normal distributions [29] (see Appendix B).

In this paper, we interpreted the closed-form formula for same-mean multivariate normal distributions in two different ways:

- First, as the Fisher–Rao geodesic midpoint of the sided Kullback–Leibler centroids. This interpretation lets us relax the midpoint definition to arbitrary exponential families to define the Jeffreys–Fisher–Rao center (the JFR center of Definition 2);
- Second, as an inductive $\left(A,m_{\nabla F}\right)$ center using a multivariate Gauss-type double sequence, which converges to the Gauss–Bregman center (the GB center of Definition 3). The latter definition yields an extension of Nakamura’s arithmetic–harmonic (A,H) mean [41] to an arbitrary $\left(A,m_{\nabla F}\right)$ mean for which we proved convergence under a separability condition in Theorem 3. Convergence proof remains to be performed in the general case, although we noticed in practice convergence when ∇F(θ) is the moment parameter of categorical or multivariate normal distributions.

In general, the Jeffreys, JFR, and GB centers differ from each other (e.g., the case of categorical distributions). But for sets of same-mean normal distributions, they remarkably coincide altogether: namely, this was the point of departure of this research. We reported generic or closed-form formulas for the JFR centers of (a) uni-order parametric families in Section 4.1 (Theorem 4), (b) categorical families in Section 4.2 (Theorem 5), and (c) multivariate normal families in Section 4.3 (Theorem 6). Table 3 summarizes the new results obtained in this paper and states references of prior work. Notice that in practice, we approximate the Gauss–Bregman center by prescribing a number of iterations T∈N for the Gauss–Bregman double sequence to obtain $m_{\mathrm{GB}}^{\left(T\right)}$. Prescribing the number of GB center iterations *T* allows us to tune the time complexity of computing $m_{\mathrm{GB}}^{\left(T\right)}$ while adjusting the quality of the approximation of the Jeffreys centroid.

In applications requiring the Jeffreys centroid, we thus propose to either use the fast Jeffreys–Fisher–Rao center when a closed-form formula is available for the family of distributions at hand or use the Gauss–Bregman center approximation with a prescribed number of iterations as a drop-in replacement of the numerical Jeffreys centroids while keeping the Jeffreys divergence (the centers we defined are not centroids as we do not exhibit distances from which they are population minimizers).

More generally, let us rephrase the results in a purely geometric setting using the framework of information geometry [14]: let $P_{1},\ldots ,P_{n}$ be a set of *n* points weighted by a vector $w\in \Delta_{n}$ on an *m*-dimensional dually flat space $\left(M,g,\nabla ,\nabla^{*}\right)$ with the ∇-affine coordinate system θ(·) and dual $\nabla^{*}$-affine coordinate system η(·), where ∇ and $\nabla^{*}$ are two torsion-free dual affine connections. The Riemannian metric *g* is a Hessian metric [54], which may be expressed in the θ-coordinate system as $g\left(\theta \right)=\nabla^{2}F\left(\theta \right)$ or in the dual coordinate system as $g\left(\eta \right)=\nabla^{2}F^{*}\left(\eta \right)$, where F(θ) and $F^{*}\left(\eta \right)$ are dual convex potential functions related by the Legendre–Fenchel transform [14,54]. Let $\eta_{i}=\nabla F\left(\theta_{i}\right)$ and $\theta_{i}=\nabla F^{*}\left(\eta_{i}\right)$ be the coordinates of point $P_{i}$ in the η- and θ-coordinate systems, respectively. An arbitrary point *P* can be either referenced in the θ-coordinate system ($P=P_{\theta }$) or in the η-coordinate system ($P=P_{\eta }$). Then, the Jeffreys–Fisher–Rao center is defined as the midpoint with respect to the Levi-Civita connection $\bar{\nabla }=\frac{\nabla +\nabla^{*}}{2}=\nabla^{g}$ of *g*:

(20) $$C_{\mathrm{JFR}}:=\gamma_{\bar{\nabla }}\left(C_{\bar{\theta }},C_{\underline{\theta }},\frac{1}{2}\right)=:C_{\bar{\theta }}\#C_{\underline{\theta }}.$$

The point $C_{\bar{\theta }}$ is the centroid with respect to the canonical flat divergence $D\left(P:Q\right)=F\left(\theta \left(P\right)\right)+F^{*}\left(\eta \left(Q\right)\right)-\sum_{i=1}^{m}\theta_{i}\left(P\right)\eta_{i}\left(Q\right)$, and the point $C_{\underline{\theta }}$ is the centroid with respect to the dual canonical flat divergence $D^{*}\left(P:Q\right):=D\left(Q:P\right)$. The canonical divergence is expressed using the mixed coordinates θ/η but can also be expressed using the θ-coordinates as an equivalent Bregman divergence $D\left(P:Q\right)=B_{F}\left(\theta \left(P\right):\theta \left(Q\right)\right)$ or as a reverse dual Bregman divergence $D\left(P:Q\right)=B_{F^{*}}\left(\eta \left(Q\right):\eta \left(P\right)\right)$. This JFR center $C_{\mathrm{JFR}}$ approximates the symmetrized centroid with respect to the canonical symmetrized divergence S(P,Q)=D(P:Q)+D(Q:P) (i.e., Jeffreys divergence when written using the θ-coordinate system). This symmetrized divergence S(P,Q) can be interpreted as the energy of the Riemannian length element ds along the primal geodesic γ(t) and dual geodesic $\gamma^{*}\left(t\right)$ (with $\gamma \left(0\right)=\gamma^{*}\left(0\right)=P$ and $\gamma \left(1\right)=\gamma^{*}\left(1\right)=Q$), see [14]: $S\left(P,Q\right)=\int_{0}^{1}ds^{2}\left(\gamma \left(t\right)\right)dt=\int_{0}^{1}ds^{2}\left(\gamma^{*}\left(t\right)\right)dt$. The Riemannian distance ρ(P,Q) corresponds to the Riemannian length element along the Riemannian geodesic $\bar{\gamma }\left(t\right)$ induced by the Levi-Civita connection $\bar{\nabla }=\frac{\nabla +\nabla^{*}}{2}$: $\rho \left(P,Q\right)=\int_{0}^{1}ds\left(\bar{\gamma }\left(t\right)\right)dt$.

**Figure 10:**
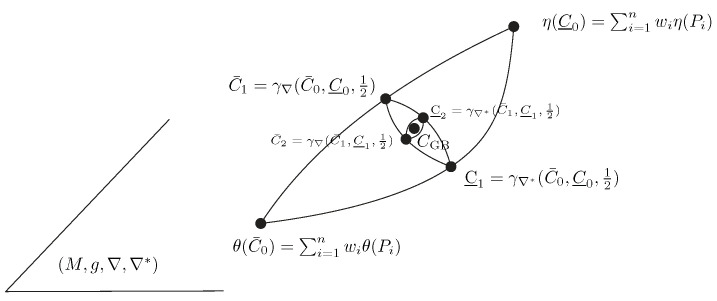
Illustration on a dually flat space of the double sequence inducing a Gauss–Bregman center in the limit.

**Figure 11:**
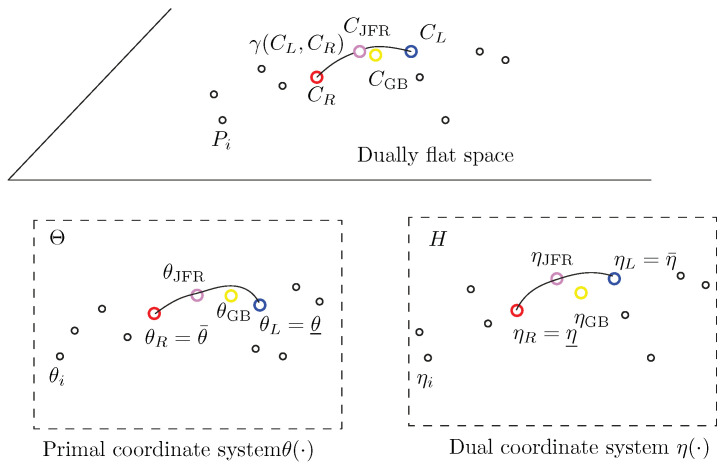
Illustration of the Jeffreys–Fisher–Rao and Gauss–Bregman centers a dually flat space. γ denotes the Riemannian geodesic.

The inductive Gauss–Bregman center $C_{\mathrm{GB}}$ is obtained as a limit sequence of taking iteratively the ∇ midpoints and $\nabla^{*}$ midpoints with respect to the ∇ and $\nabla^{*}$ connections. Those midpoints correspond to the right and left centroids $C_{t+1}$ and $C_{t+1}^{*}$ with respect to D(·:·):

$$\begin{array}{ccc} C_{t+1} & = & \gamma_{\nabla }\left(C_{t},C_{t}^{*},\frac{1}{2}\right),\\ C_{t+1}^{*} & = & \gamma_{\nabla^{*}}\left(C_{t},C_{t}^{*},\frac{1}{2}\right), \end{array}$$

initialized with $\theta \left(C_{0}\right)=\sum_{i=1}^{n}w_{i}\theta \left(P_{i}\right)$ and $\eta \left(C_{0}^{*}\right)=\sum_{i=1}^{n}w_{i}\eta \left(P_{i}\right)$. We have $C_{0}=\arg\min_{C\in M}$$\sum_{i}w_{i}D\left(P_{i}:C\right)$ and $C_{0}^{*}=\arg\min_{C\in M}\sum_{i}w_{i}D\left(P_{i}:C\right)$. Figure 10 geometrically illustrates the double sequence of iteratively taking dual geodesic midpoints to converge toward the Gauss–Bregman center $C_{\mathrm{GB}}$. Thus, the GB double sequence can be interpreted as a geometric optimization technique. Figure 11 illustrates the JFR and GB centers on a dually flat space. Notice that $C_{\mathrm{JFR}}$ has coordinates ${\mathrm{JFR}}_{F}\left(P_{\theta };w\right)$ in the θ-chart and coordinates ${\mathrm{JFR}}_{F^{*}}\left(P_{\eta };w\right)$ in the η-chart. Similarly, $C_{\mathrm{GB}}$ has coordinates ${\mathrm{GB}}_{F}\left(\bar{\theta },\underline{\theta }\right)$ in the θ-chart and coordinates ${\mathrm{GB}}_{F^{*}}\left(\bar{\eta },\underline{\eta }\right)$ in the η-chart.

As a final remark, let us emphasize that choosing a proper mean or center depends on the application at hand [55,56]. For example, in Bayesian hypothesis testing, the Chernoff mean [57] is used to upper bound Bayes’ error and has been widely used in information fusion [18] for its empirical robustness [58] in practice. Jeffreys centroid has been successfully used in information retrieval tasks [6].

## Figures and Tables

**Table 1:** Experiments for JFR and GB centers approximating the numerical Jeffreys centroid.

dim.	Jeffreys–Fisher–Rao Center						Gauss–Bregman Center					
	Avg Info ϵ	Max Info ϵ	Avg TV	Max TV	Avg Time	×Speed	Avg Info ϵ	Max Info ϵ	Avg TV	Max TV	Avg Time	×Speed
d = 2	5.662 × $10^{-6}$	6.386 × $10^{-3}$	8.735 × $10^{-5}$	5.005 × $10^{-2}$	1.614 × $10^{-7}$	82.541	1.507 × $10^{-4}$	9.745 × $10^{-2}$	6.304 × $10^{-4}$	5.005 × $10^{-2}$	5.072 × $10^{-7}$	26.258
d = 4	1.283 × $10^{-5}$	5.294 × $10^{-3}$	1.690 × $10^{-4}$	3.969 × $10^{-2}$	1.418 × $10^{-7}$	182.309	4.696 × $10^{-4}$	7.695 × $10^{-2}$	1.431 × $10^{-3}$	3.969 × $10^{-2}$	1.623 × $10^{-7}$	159.304
d = 8	2.766 × $10^{-5}$	6.970 × $10^{-3}$	2.210 × $10^{-4}$	3.470 × $10^{-2}$	1.772 × $10^{-7}$	292.125	1.011 × $10^{-3}$	9.677 × $10^{-2}$	2.033 × $10^{-3}$	3.470 × $10^{-2}$	1.955 × $10^{-7}$	264.680
d = 16	3.531 × $10^{-5}$	8.544 × $10^{-3}$	2.325 × $10^{-4}$	2.450 × $10^{-2}$	6.318 × $10^{-7}$	224.370	1.388 × $10^{-3}$	9.231 × $10^{-2}$	2.275 × $10^{-3}$	2.450 × $10^{-2}$	7.208 × $10^{-7}$	196.660
d = 32	4.123 × $10^{-5}$	5.242 × $10^{-3}$	2.457 × $10^{-4}$	1.230 × $10^{-2}$	4.811 × $10^{-7}$	462.754	1.674 × $10^{-3}$	5.398 × $10^{-2}$	2.449 × $10^{-3}$	1.230 × $10^{-2}$	5.457 × $10^{-7}$	408.007
d = 64	4.747 × $10^{-5}$	3.437 × $10^{-3}$	2.486 × $10^{-4}$	9.756 × $10^{-3}$	9.789 × $10^{-7}$	578.354	1.863 × $10^{-3}$	3.685 × $10^{-2}$	2.498 × $10^{-3}$	9.756 × $10^{-3}$	1.160 × $10^{-6}$	488.246
d = 128	5.020 × $10^{-5}$	2.540 × $10^{-3}$	2.491 × $10^{-4}$	6.580 × $10^{-3}$	5.874 × $10^{-6}$	477.412	1.937 × $10^{-3}$	2.374 × $10^{-2}$	2.522 × $10^{-3}$	6.580 × $10^{-3}$	6.605 × $10^{-6}$	424.609
d = 256	4.735 × $10^{-5}$	1.410 × $10^{-3}$	2.476 × $10^{-4}$	4.855 × $10^{-3}$	9.349 × $10^{-6}$	528.452	1.914 × $10^{-3}$	1.521 × $10^{-2}$	2.529 × $10^{-3}$	4.855 × $10^{-3}$	1.110 × $10^{-5}$	445.304

**Table 2:** Experiments for JFR and GB centers approximating the numerical Jeffreys centroid for the following setting of two normalized histograms of 3 bins: $\left(\frac{1}{3},\frac{1}{3},\frac{1}{3}\right)$ and (1−α,α/2,α/2).

α	Info. ϵ	TV ϵ	Avg Time	×Speed	Info. ϵ	TV ϵ	Avg Time	×Speed
1.000 × $10^{-1}$	6.882 × $10^{-9}$	2.495 × $10^{-5}$	1.767 × $10^{-7}$	125.960	1.338 × $10^{-6}$	3.480 × $10^{-4}$	2.334 × $10^{-7}$	95.356
1.000 × $10^{-2}$	2.607 × $10^{-5}$	1.722 × $10^{-3}$	1.371 × $10^{-7}$	167.932	1.061 × $10^{-3}$	1.108 × $10^{-2}$	1.565 × $10^{-7}$	147.104
1.000 × $10^{-3}$	6.262 × $10^{-4}$	7.530 × $10^{-3}$	1.033 × $10^{-7}$	218.450	1.272 × $10^{-2}$	3.534 × $10^{-2}$	1.208 × $10^{-7}$	186.698
1.000 × $10^{-4}$	3.632 × $10^{-3}$	1.570 × $10^{-2}$	1.171 × $10^{-7}$	193.345	4.580 × $10^{-2}$	6.065 × $10^{-2}$	1.367 × $10^{-7}$	165.571
1.000 × $10^{-5}$	1.121 × $10^{-2}$	2.419 × $10^{-2}$	1.546 × $10^{-7}$	150.807	7.322 × $10^{-3}$	1.929 × $10^{-2}$	2.834 × $10^{-7}$	82.261
1.000 × $10^{-6}$	2.457 × $10^{-2}$	3.204 × $10^{-2}$	1.619 × $10^{-7}$	141.896	1.655 × $10^{-2}$	2.579 × $10^{-2}$	2.512 × $10^{-7}$	91.467
1.000 × $10^{-7}$	4.375 × $10^{-2}$	3.897 × $10^{-2}$	1.357 × $10^{-7}$	170.065	3.065 × $10^{-2}$	3.183 × $10^{-2}$	2.131 × $10^{-7}$	108.314
1.000 × $10^{-8}$	6.806 × $10^{-2}$	4.492 × $10^{-2}$	1.315 × $10^{-7}$	173.698	4.948 × $10^{-2}$	3.725 × $10^{-2}$	2.017 × $10^{-7}$	113.292
1.000 × $10^{-9}$	9.651 × $10^{-2}$	4.999 × $10^{-2}$	1.125 × $10^{-7}$	208.627	7.240 × $10^{-2}$	4.199 × $10^{-2}$	1.590 × $10^{-7}$	147.610
1.000 × $10^{-10}$	1.281 × $10^{-1}$	5.428 × $10^{-2}$	8.366 × $10^{-8}$	242.967	9.862 × $10^{-2}$	4.610 × $10^{-2}$	1.111 × $10^{-7}$	183.000
1.000 × $10^{-11}$	1.621 × $10^{-1}$	5.792 × $10^{-2}$	1.066 × $10^{-7}$	215.817	1.274 × $10^{-1}$	4.963 × $10^{-2}$	1.325 × $10^{-7}$	173.614
1.000 × $10^{-12}$	1.979 × $10^{-1}$	6.100 × $10^{-2}$	1.028 × $10^{-7}$	229.484	1.580 × $10^{-1}$	5.266 × $10^{-2}$	1.329 × $10^{-7}$	177.581
1.000 × $10^{-13}$	2.348 × $10^{-1}$	6.363 × $10^{-2}$	9.541 × $10^{-8}$	244.587	1.901 × $10^{-1}$	5.526 × $10^{-2}$	1.255 × $10^{-7}$	185.940
1.000 × $10^{-14}$	2.727 × $10^{-1}$	6.589 × $10^{-2}$	1.062 × $10^{-7}$	219.787	2.231 × $10^{-1}$	5.750 × $10^{-2}$	1.361 × $10^{-7}$	171.456
1.000 × $10^{-15}$	3.112 × $10^{-1}$	6.784 × $10^{-2}$	9.043 × $10^{-8}$	248.688	2.570 × $10^{-1}$	5.943 × $10^{-2}$	1.322 × $10^{-7}$	170.122
1.000 × $10^{-16}$	3.483 × $10^{-1}$	6.946 × $10^{-2}$	8.857 × $10^{-8}$	267.219	2.897 × $10^{-1}$	6.105 × $10^{-2}$	1.438 × $10^{-7}$	164.535

**Table 3:** Summary of the results: Δ indicates a generic formula, √ a closed-form formula, and × no-known formula.

Family	Jeffreys	Jeffreys–Fisher–Rao	Gauss–Bregman
Exponential family	Equation (2)	Definition 2	Definition 3
One-dimensional exponential family	×	Δ	×
	Theorem 4		[43]
Categorical family	Δ	√	×
	[31]	Theorem 5	Theorem 3
Normal family	×	√	×
	[28]	Theorem 6	Theorem 3
Centered normal family	√	√	√
	[29]	[29]	[41]

## Data Availability

No new data were created or analyzed in this study. Data sharing is not applicable to this article.

## Appendix A. Numerical Jeffreys Centroids for Categorical Distributions

Algorithm A1 implements the method described in [31] for numerically finely approximating the Jeffreys centroid of a weighted set of categorical distributions.

**Algorithm A1:** Numerical approximation of the SKL/Jeffreys centroid for categorical distributions.

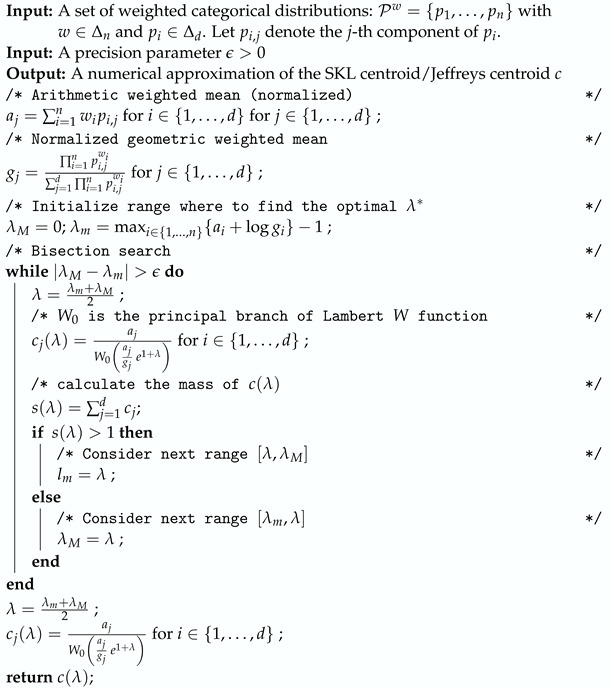

## Appendix B. Closed-Form Formula for the Symmetrized Log Det Centroids

Consider a set $P=\{P_{1},\ldots ,P_{n}\}$ of *n* symmetric positive-definite matrices of the *d*-dimensional SPD cone ${\mathrm{Sym}}^{++}\left(d,R\right)$ weighted by a vector $w=\left(w_{1},\ldots ,w_{n}\right)\in \Delta_{n}^{\circ }$ such that $P_{i}$ has weight $w_{i}$ for i∈{1,…,n}. The log det divergence [59] is a Bregman divergence induced by the strictly convex and differential generator $F_{\mathrm{ld}}\left(X\right)=-\log\det\left(X\right)$ on ${\mathrm{Sym}}^{++}\left(d,R\right)$ equipped with the inner product 〈X,Y〉=tr(XY) for X,Y∈Sym(d,R):

(A1) $$D_{\mathrm{ld}}\left(X:Y\right)=B_{F_{\mathrm{ld}}}\left(X:Y\right)=F\left(X\right)-F\left(Y\right)-\langle X-Y,\nabla F_{\mathrm{ld}}\left(Y\right)\rangle .$$

Since we have $\nabla F_{\mathrm{ld}}\left(X\right)=-\nabla \log\det\left(X\right)=-\frac{\nabla \det(X)}{\det(X)}=-{\left(X^{-1}\right)}^{⊤}$ (hence $\nabla F_{\mathrm{ld}}\left(X\right)=-X^{-1}$ for symmetric matrices), it follows that the log det divergence is

$$\begin{array}{ccc} D_{\mathrm{ld}}\left(X:Y\right) & = & \log\det\left(YX^{-1}\right)+\mathrm{tr}\left(\left(X-Y\right)Y^{-1}\right),\\ & = & \mathrm{tr}\left(XY^{-1}\right)-\log\det\left(XY^{-1}\right)-d, \end{array}$$

using the properties that det(X)det(Y)=det(XY), $\det\left(X^{-1}\right)=\frac{1}{\det(X)}$, and tr(I)=d where *I* denotes the d×d identity matrix. When d=1, we recover the Itakura–Saito divergence [45] obtained for $F_{\mathrm{IS}}\left(x\right)=-\log x$ (Burg negative entropy) with $F_{\mathrm{IS}}^{\prime }\left(x\right)=-\frac{1}{x}$:

$$D_{\mathrm{IS}}\left(x:y\right)=B_{F_{\mathrm{IS}}}\left(x:y\right)=\frac{x}{y}-\log\frac{x}{y}-1,\;x,y>0.$$

The log det divergence is known in statistics as Stein’s loss [37,38] and has been used for estimating covariance matrices. The log det divergence $S_{\mathrm{ld}}$ satisfies the following invariance properties:

- Inversion invariance: $S_{\mathrm{ld}}\left(X^{-1},Y^{-1}\right)=S_{\mathrm{ld}}\left(X,Y\right)$;
- Congruence invariance: for any invertible matrix A∈GL(d), we have $S_{\mathrm{ld}}\left(AXA^{⊤},AY^{-1}A^{⊤}\right)=S_{\mathrm{ld}}\left(X,Y\right)$.

The Jeffreys’ symmetrized log det divergence (SLD) is thus

(A2) $$\begin{array}{ccc} S_{\mathrm{ld}}\left(X,Y\right) & = & D_{\mathrm{ld}}\left(X:Y\right)+D_{\mathrm{ld}}\left(Y:X\right)=\mathrm{tr}\left(\left(Y^{-1}-X^{-1})(X-Y\right)\right), \end{array}$$

(A3) $$\begin{array}{ccc} & = & \mathrm{tr}\left(X^{-1}Y+Y^{-1}X-2I\right). \end{array}$$

When d=1, the SLD corresponds to the COSH distance [60] (COSine Hyperbolic distance, the symmetrized Itakura–Saito divergence) when d=1:

$$D_{\mathrm{COSH}}\left(x:y\right)=\left(\frac{y}{x}-\frac{1}{x}\right)=\frac{x}{y}+\frac{y}{x}-2.$$

Consider a family $N_{\mu }=\left\{p_{\mu ,\sum_{1}},\ldots ,p_{\mu ,\sum_{n}}\right\}$ of *n* multivariate normal distributions centered at the same mean $\mu \in R^{d}$ with covariance matrices $\sum_{1},\ldots ,\sum_{n}$. The set of same-mean normal distributions forms an exponential family with the natural parameter $\theta =\sum^{-1}$ (the precision matrix) corresponding to the sufficient statistics $t\left(x\right)=-\frac{1}{2}xx^{⊤}$, and the log-normalizer $F\left(\theta \right)=-\frac{1}{2}\log\det\left(\theta \right)$. Thus, the Kullback–Leibler divergence between $p_{\mu ,\sum_{i}}$ and $p_{\mu ,\sum_{j}}$ corresponds to a log det divergence [16]:

$$D_{\mathrm{KL}}\left[p_{\mu ,\sum_{i}},p_{\mu ,\sum_{j}}\right]=B_{F}\left(\theta_{j}:\theta_{i}\right)=D_{\mathrm{ld}}\left(\sum_{j}^{-1}:\sum_{i}^{-1}\right),$$

and therefore the Jeffreys divergence $D_{J}\left[p_{\mu ,\sum_{i}},p_{\mu ,\sum_{j}}\right]$ corresponds to the matrix COSH/symetrized log-det divergence:

(A4) $$D_{J}\left[p_{\mu ,\sum_{i}},p_{\mu ,\sum_{j}}\right]=S_{\mathrm{ld}}\left(\sum_{i}^{-1},\sum_{j}^{-1}\right)=\mathrm{tr}\left(\left(\sum_{i}^{-1}-\sum_{j}^{-1})(\sum_{j}-\sum_{i}\right)\right).$$

The left KL centroid corresponds to a right Bregman centroid on the natural parameters (the center of mass of the natural parameters), which corresponds to a weighted matrix harmonic mean on the covariance matrices:

$$C_{L}^{\mathrm{KL}}=C_{R}^{B_{F}}={\left(\sum_{i=1}^{n}w_{i}\sum_{i}^{-1}\right)}^{-1}.$$

The right KL centroid is a left Bregman centroid (i.e., a quasi-arithmetic mean for $h\left(X\right)=-X^{-1}$ with $h^{-1}\left(Y\right)=-Y^{-1}$) which corresponds to the inverse of the weighted arithmetic mean on the covariance matrices:

$$C_{R}^{\mathrm{KL}}=C_{L}^{B_{F}}={\left(\sum_{i=1}^{n}w_{i}\sum_{i}\right)}^{-1}.$$

We state the remarkable case of the closed-form formula for the symmetrized Bregman logdet centroid:

**Proposition** **A1** ([29])**.** *The symmetrized log det centroid of a set $P^{w}=\left\{\left(w_{i},P_{i}\right)\right\}$ of n weighted positive-definite matrices is A#H where $A=\sum_{i}w_{i}P_{i}$ and $H={\left(\sum_{i}w_{i}P_{i}^{-1}\right)}^{-1}$ are the weighted arithmetic and harmonic means and A#B is the matrix geometric mean.*

Since the proof was only briefly sketched in [29], we report a full-length proof for the sake of completeness:

**Proof.** We have

$$\min_{X}\sum_{i}w_{i}S_{\mathrm{ld}}\left(X,P_{i}\right)\equiv \min_{X}\mathrm{tr}\left(X^{-1}A+H^{-1}X\right).$$

Setting the gradient of the right-hand-side term to zero using matrix calculus [61] yields

$$\begin{array}{c} \nabla_{X}\mathrm{tr}\left(X^{-1}A+H^{-1}X\right)=\mathrm{tr}\left(\nabla_{X}\left(X^{-1}A+H^{-1}X\right)\right)=0. \end{array}$$

Using the matrix calculus property that $\nabla \left(M^{-1}\right)=-M^{-1}\left(\nabla M\right)M^{-1}$ for $M=X^{-1}A$, we obtain

$$X^{-1}AX^{-1}-H^{-1}=0.$$

That is, we need to solve the following Ricatti equation:

$$X^{-1}AX^{-1}=H^{-1}.$$

The well-known Ricatti equation $XA^{-1}X=B$ solves [40] as X=A#B, and therefore we obtain

$$X^{-1}=A^{-1}\#H^{-1}.$$

Finally, we use the invariance property of the geometric mean under matrix inversion, $A^{-1}\#H^{-1}=A\#H$, to obtain the result $C_{S}^{\mathrm{ld}}=A\#H$. □

The Riemannian Hessian metric g(θ) induced by $F\left(\theta \right)=-\frac{1}{2}\log\det\left(\theta \right)$ is

$$g_{\theta }\left(S_{1},S_{2}\right)=\mathrm{tr}\left(\theta^{-1}S_{1}\theta^{-1}S_{2}\right),$$

where $S_{1}$ and $S_{2}$ are two symmetric matrices of the tangent space $T_{\theta }$ at θ. The metric tensor *g* is commonly called the trace metric or Affine-Invariant Riemannian Metric (AIRM) [62].

It follows that the Riemannian geodesic midpoint is the matrix geometric mean [63] given by

$$X\#Y=X^{\frac{1}{2}}\;{\left(X^{-\frac{1}{2}}\;Y\;X^{-\frac{1}{2}}\right)}^{\frac{1}{2}}\;X^{\frac{1}{2}}.$$

We have ρ(X,X#Y)=ρ(X#Y,Y), where ρ(·,·) denotes the geodesic length distance on the Riemannian manifold. The geodesic length is given by the following formula [64,65]:

$$\rho \left(P_{1},P_{2}\right)={∥\log\left(P_{1}^{-\frac{1}{2}}\;P_{2}\;P_{1}^{-\frac{1}{2}}\right)∥}_{F}=\sqrt{\sum_{i=1}^{d}\log^{2}\lambda_{i}\left(P_{1}^{-\frac{1}{2}}\;P_{2}\;P_{1}^{-\frac{1}{2}}\right)},$$

where the $\lambda_{i}\left(X\right)$s are the generalized eigenvalues of *X*.

We state the theorem geometrically characterizing the Jeffreys centroid of a weighted set of centered multivariate normal distributions.

**Theorem** **A1** (The Jeffreys centroid of *n* weighted centered multivariate normal distributions)**.** *The Jeffreys centroid $C_{S}$ of a weighted set $\left\{p_{\mu ,\sum_{i}}\right\}$ of centered normal distributions $N(\mu ,\sum_{i})$ with a weighted $w\in \Delta_{n}$ corresponds to the midpoint of the Fisher–Rao geodesic linking the left and right SKL centroids:*

(A5) $$C_{S}=\left(\sum_{i=1}^{n}w_{i}\sum_{i}\right)\#{\left(\sum_{i=1}^{n}w_{i}\sum_{i}^{-1}\right)}^{-1},$$

*where X#Y is the geometric matrix mean:*

$$X\#Y=X^{\frac{1}{2}}\;{\left(X^{-\frac{1}{2}}\;Y\;X^{-\frac{1}{2}}\right)}^{\frac{1}{2}}\;X^{\frac{1}{2}}.$$

This result first appeared in [29] (Lemma 17.4.3, item 3) and also appeared in an indirect but more general form in [66] (Theorem 5.3). Indeed, in [66], the authors define the regularized symmetric log det divergence as follows:

$$S_{\mathrm{ld}}^{\epsilon }\left(X,Y\right)=\mathrm{tr}\left(\left(X-Y\right)\left({\left(Y+\epsilon I\right)}^{-1}-{\left(X+\epsilon I\right)}^{-1}\right)\right),\;\epsilon >0.$$

This extended definition of the symmetrized logdet divergence allows one to consider degenerate semi-positive definite matrices.

## Appendix C. Fisher–Rao Midpoint for Multivariate Normal Distributions

The expression of the Fisher–Rao geodesics for multivariate normal distributions (MVNs) passing through two given *d*-variate normal distributions was elucidated in [32]. We give below the method for finding those Fisher–Rao MVN midpoints without the underlying geometric explanation that relies on a Riemannian submersion in dimension 2d+1 [32]. The Python software library pyBregMan [52] provides an implementation of those Fisher–Rao MVN midpoints.

Fisher–Rao geodesic midpoint N=N(μ,∑) of $N_{0}=N\left(\mu_{0},\sum_{0}\right)$ and $N_{1}=N\left(\mu_{1},\sum_{1}\right)$ - For i∈{0,1}, let $G_{0}=M_{0}\;D_{0}\;M_{0}^{⊤}$ and $G_{1}=M_{1}\;D_{1}\;M_{1}^{⊤}$, where $$\begin{array}{ccc} D_{0} & = & \left[\begin{array}{ccc} \sum_{0}^{-1} & 0 & 0\\ 0 & 1 & 0\\ 0 & 0 & \sum_{0} \end{array}\right],\\ M_{0} & = & \left[\begin{array}{ccc} I_{d} & 0 & 0\\ \mu_{0}^{⊤} & 1 & 0\\ 0 & -\mu_{0} & I_{d} \end{array}\right],\\ D_{1} & = & \left[\begin{array}{ccc} \sum_{1}^{-1} & 0 & 0\\ 0 & 1 & 0\\ 0 & 0 & \sum_{1} \end{array}\right],\\ M_{1} & = & \left[\begin{array}{ccc} I_{d} & 0 & 0\\ \mu_{1}^{⊤} & 1 & 0\\ 0 & -\mu_{1} & I_{d} \end{array}\right], \end{array}$$ where $I_{d}$ denotes the identity matrix of shape d×d. That is, matrices $G_{0}$ and $G_{1}\in {\mathrm{Sym}}_{+}\left(2d+1,R\right)$ can be expressed by block Cholesky factorizations. - Consider the Riemannian geodesic midpoint *G* in ${\mathrm{Sym}}_{+}\left(2d+1,R\right)$ with respect to the trace metric: $$G=G_{0}^{\frac{1}{2}}\;{\left(G_{0}^{-\frac{1}{2}}G_{1}G_{0}^{-\frac{1}{2}}\right)}^{\frac{1}{2}}\;G_{0}^{\frac{1}{2}}.$$ In order to compute the matrix power $G^{p}$ for p∈R, we first calculate the Singular Value Decomposition (SVD) of *G*: $G=O\;L\;O^{⊤}$ (where *O* is an orthogonal matrix and $L=\mathrm{diag}(\lambda_{1},\ldots ,\lambda_{2d+1})$ a diagonal matrix) and then obtain the matrix power as $G^{p}=O\;L^{p}\;O^{⊤}$ with $L^{p}=\mathrm{diag}\left(\lambda_{1}^{p},\ldots ,\lambda_{2d+1}^{p}\right)$. - Retrieve N=N(μ,∑) from matrix *G*: $$\begin{array}{ccc} \sum & = & {\left[G\right]}_{1:d,1:d}^{-1},\\ \mu & = & \sum \;{\left[G\right]}_{1:d,d+1}, \end{array}$$ where ${\left[G\right]}_{1:d,1:d}$ denotes the block matrix with rows and columns ranging from one to *d* extracted from the (2d+1)×(2d+1) matrix *G*, and ${\left[G\right]}_{1:d,d+1}$ is similarly the column vector of $R^{d}$ extracted from *G*.
